# Diffusion models in robotics: a comprehensive review

**DOI:** 10.3389/frobt.2026.1905499

**Published:** 2026-09-29

**Authors:** Linxin Bai, Chenkang Du, Jinfang Wang, Xinrong Chen

**Affiliations:** 1 College of Biomedical Engineering, Fudan University, Shanghai, China; 2 Shanghai Key Laboratory of Medical Image Computing and Computer Assisted Intervention, Fudan University, Shanghai, China; 3 General Hospital of Ningxia Medical University, Yinchuan, China

**Keywords:** diffusion models, imitation learning, reinforcement learning, robotics, scaling up robotics data, task planning

## Abstract

Diffusion models are a powerful class of generative models that emerge in recent years to transform Gaussian noise into samples of the target distribution through an iterative denoising process. Due to the high training stability and powerful generative capabilities, diffusion models have surpassed previous generative models and demonstrate potential applications in the field of robotics. In the past few years, this area has gained increasing attention, and the number of studies applying diffusion models to the field of robotics has grown exponentially. This review aims to provide an overview of this emerging field, helping researchers understand the current state of development, with the hope of inspiring new research directions. First, we overview the foundation of diffusion models along with their development in recent years. On this basis, we provide an overview of the application of diffusion modeling in robotics from four macro dimensions: environmental data preparation, decision-making control paradigms including reinforcement and imitation learning, high-level cognitive reasoning for task planning, and specialized mechanical or sensory applications. We discuss and summarize the innovations, contributions, and limitations of these works. We then discuss the limitations and challenges faced by the field in terms of safety issues, real-time inference and model size, simulation to the real world gap, datasets and unified benchmarks, and embodied foundation models. Finally, we summarize the review and provide an insight into future research directions.

## 1 Introduction

As a new paradigm of artificial intelligence (AI), generative artificial intelligence (GAI) has received a great deal of attention in recent years. In contrast to traditional AI, GAI uses a large amount of data to train a generative model, which then outputs new data with a similar distribution to the training data. The generated data encompasses a diverse range of modalities, such as text, image, and video. Due to the promising application prospects, generative models such as GPT (Radford et al., 2018), DALL-E (Ramesh et al., 2022), and Sora (Brooks et al., 2024) have gained widespread attention. With remarkable success, GAI is applied extensively in industry, healthcare, education, and other domains. Therefore, researchers have also generated interest and ideas for applying GAI to the field of robotics. Despite Variational Autoencoders (VAEs) or Generative Adversarial Networks (GANs) are attempted for the applications (Wang and Meger, 2021; Zhan et al., 2021; Huang et al., 2017; Imre, 2021; Moon and Seo, 2019) in robotics, these models have not advanced due to their limitations in terms of generative capacity and training stability.

Diffusion models are a powerful class of generative models that transform Gaussian noise into samples of the target distribution through iterative denoising processes. These models have been widely used in signal processing, image generation, and image restoration due to their powerful generation ability and solid training stability. In order to capitalize on the potent generative powers of diffusion models and advance the field of robotics, an increasing amount of research in recent years has concentrated on integrating diffusion models with robotics, as shown in Figure 1.

**FIGURE 1:**
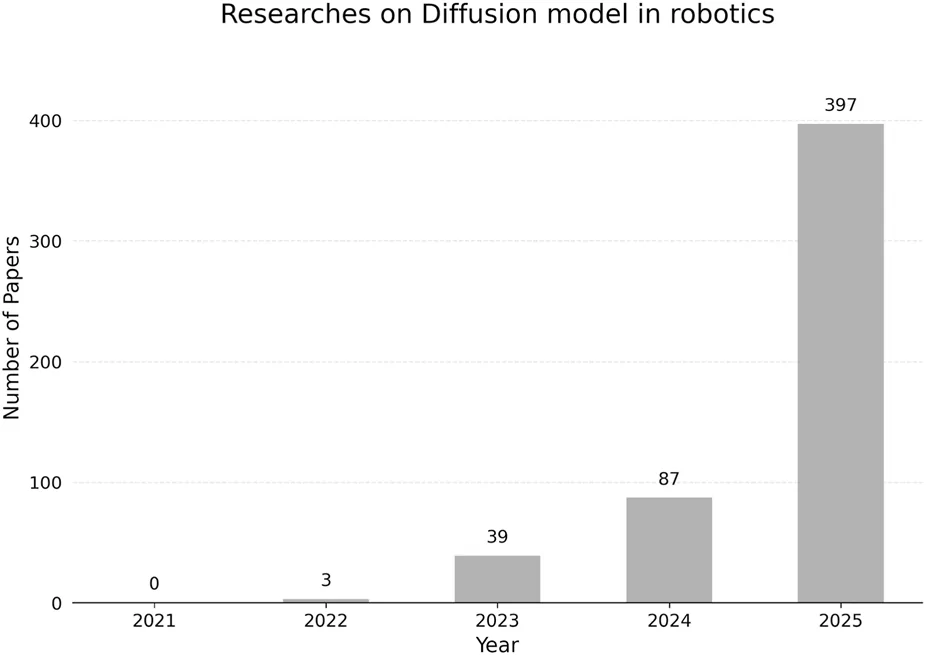
Annual trend of publications applying diffusion models to robotics (2020–2025). Data is retrieved from the ArXiv database using a conjunctive query of keywords: ‘Diffusion Models’ and ‘Generative Models’ and ‘Robotics’.

But, due to the diverse applications of diffusion models in the field of robotics, it remains challenging to fully understand how the models promote the development of robot learning in recent years. Therefore, a comprehensive review is conducted to fully investigate the practical applications of diffusion models in robotics. While recent surveys (Wolf et al., 2025; Song et al., 2025) have provided valuable and structured overviews of diffusion models in robotics, our work distinguishes itself through its comprehensive system-level pipeline scope and a granular, problem-oriented taxonomy. Prior overviews primarily classify the literature around horizontal deep learning components, organizing papers by input data modalities, neural network backbones, and general training strategies (Song et al., 2025). In contrast, our survey maps directly onto the sequential operational pipeline of autonomous robots, tracking the continuous process from environmental data preparation and decision control paradigms to high-level task reasoning. Crucially, rather than grouping methodologies into broad, monolithic categories, our framework deconstructs each domain based on the specific algorithmic and programmatic bottlenecks they resolve. This fine-grained, problem-driven classification provides a highly structured index that substantially complements and expands upon the existing component-focused review literature.

This paper is organized as follows: Section 2 describes the fundamental ideas of diffusion models and its latest developments. Section 3 provides a comprehensive overview of the applications of diffusion models in robotics by collecting and surveying research on the topic over the previous 3 years, including scaling up robotics data, RL, IL, task planning and reasoning, and other applications. Section 4 analyses the limitations of applying diffusion models in robotics, addressing the challenges that are likely to arise in the future as well as potential research directions. And finally, we provide a summary of the key points discussed in this paper.

## 2 Overview of diffusion model

Diffusion models are probabilistic generative models that utilize variational inference to construct a Markov chain. Through an iterative denoising process guided by a mean squared error (MSE) loss function, they progressively transform Gaussian noise into samples from a target distribution. Given their capacity to model complex, highly non-linear distributions, these frameworks have achieved widespread success in 2D image, video, and text generation. This section introduces the fundamental principles of diffusion models using the Denoising Diffusion Probabilistic Model (DDPM) (Ho et al., 2020) as a representative baseline, followed by a concise review of recent algorithmic advancements crucial for robotic deployment. Figure 2 provides a visual overview of these foundational frameworks, illustrating the standard DDPM alongside its score-based, conditional, and latent variants.

**FIGURE 2:**
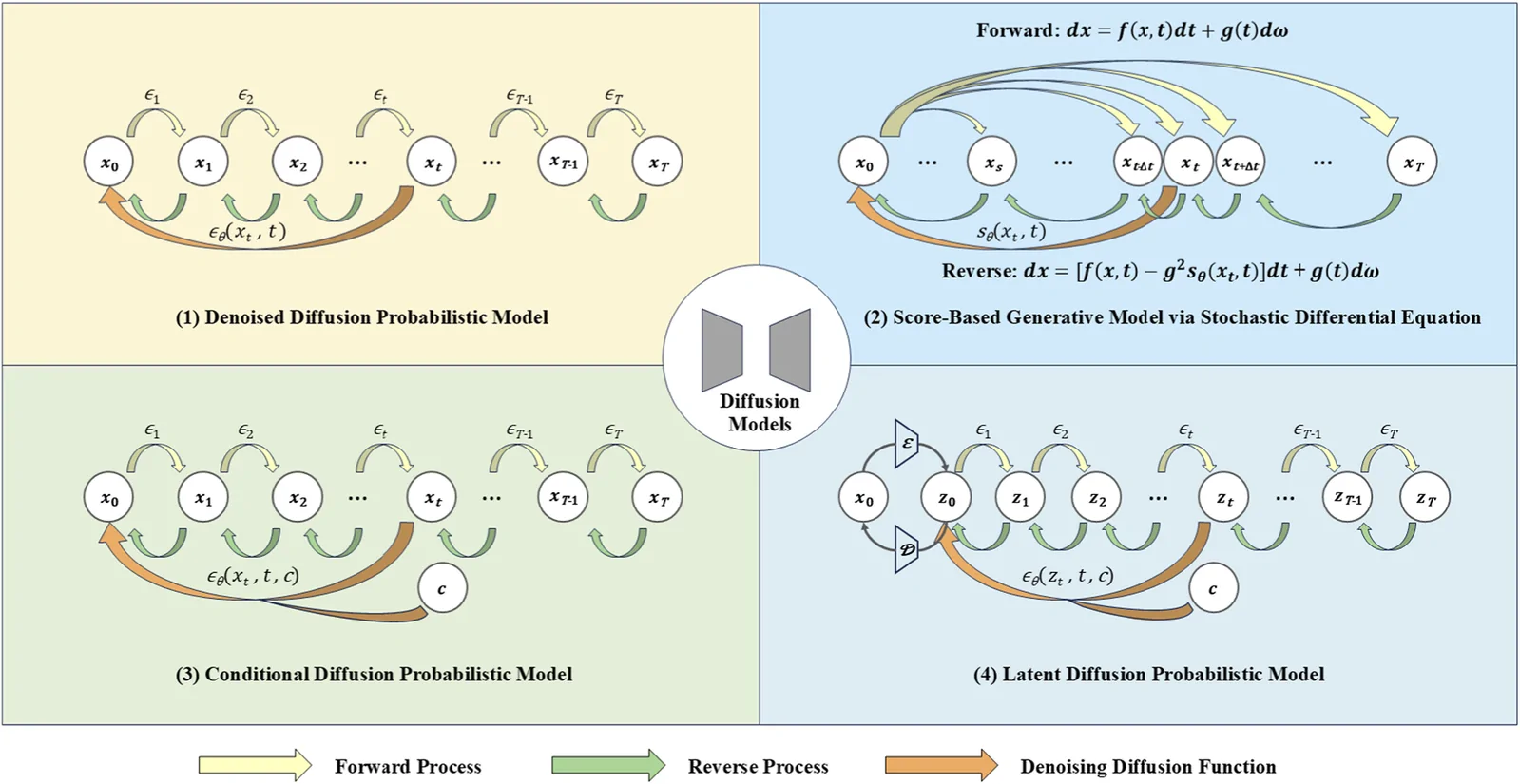
Overview of Diffusion Models. (1) The diffusion process in DDPM is implemented through iterative noise addition and removal. (2) Score-Based Generative Model governs the diffusion process through SDEs over continuous timeline (3) Conditional Diffusion Probabilistic Model employs condition c in each sampling step. (4) Latent Diffusion Probabilistic Model implements the diffusion process in a low-dimensional latent space.

### 2.1 Denoising diffusion probabilistic models

The core mathematical formulation of modern diffusion models builds upon foundational concepts introduced in 2015 (Sohl-Dickstein et al., 2015), which defined a forward process that diffuses a complex data distribution into a simpler one, alongside a learnable reverse mapping. Another critical pillar is the Score-based Generative Model (Song and Ermon, 2019), which trains a neural network to estimate the score function—defined as the gradient of the log-density of perturbed data. By perturbing data with varying noise magnitudes, score-based models generate denoised samples, establishing the theoretical bedrock for the discrete DDPM framework (Ho et al., 2020).

Formulated as a parameterized Markov chain, DDPM generates target data distributions via iterative denoising during inference, consisting of two main phases: the forward process and the reverse process.

#### 2.1.1 Forward Process

The forward process is a Markov chain that systematically introduces Gaussian noise to the uncorrupted training data $x_{0}∼q\left(x_{0}\right)$ over T steps until the data is completely degraded. The latent variables $x_{1},\ldots ,x_{T}$ are generated according to the Markovian process defined in Equations 1, 2:

$$q\left(x_{1:T}|x_{0}\right)≔\overset{T}{\underset{t=1}{\prod }}q\left(x_{t}|x_{t-1}\right)$$ (1)

$$q\left(x_{t}|x_{t-1}\right)≔N\left(x_{t};\sqrt{1-\beta_{t}}x_{t-1},\beta_{t}I\right)$$ (2)

where $\beta_{t}$ defines the variance schedule. A key property of this formulation is the ability to sample $x_{t}$ at any arbitrary timestep t in closed form as shown in Equation 3:

$$q\left(x_{t}|x_{0}\right)≔N\left(x_{t};\sqrt{{\bar{\alpha }}_{t}}x_{0},\left(1-{\bar{\alpha }}_{t}\right)I\right)$$ (3)

where $\alpha_{t}=1-\beta_{t}$ and ${\bar{\alpha }}_{t}=\prod_{i=1}^{t}\alpha_{i}$ follows the standard notation introduced by (Ho et al., 2020).

#### 2.1.2 Reverse Process

To generate new samples from pure noise, a reverse process is optimized using learnable Gaussian kernels parameterized by θ , as defined in Equation 4:

$$p_{\theta }\left(x_{t-1}|x_{t}\right)≔N\left(x_{t-1};\mu_{\theta }\left(x_{t},t\right),\Sigma_{\theta }\left(x_{t},t\right)\right)$$ (4)

where $\mu_{\theta }$ and $\Sigma_{\theta }$ denote the predicted mean and covariance. The training objective minimizes the variational lower bound (ELBO) of the negative log-likelihood, as defined in Equation 5:

$$\begin{array}{cc} L_{vlb}= & D_{\text{KL}}\left(q\left(x_{T}|x_{0}\right)\|\pi \left(x_{T}\right)\right)-\log p_{\theta }\left(x_{0}|x_{1}\right)\\ & +\underset{t>1}{\sum }D_{\text{KL}}\left(q\left(x_{t-1}|x_{t},x_{0}\right)\|p_{\theta }\left(x_{t-1}|x_{t}\right)\right) \end{array}$$ (5)

where the terms represent the prior loss, reconstruction loss, and a sequence of Kullback-Leibler (KL) divergences aligning the reverse transitions with the tractable forward posterior $q\left(x_{t-1}|x_{t},x_{0}\right)$ . Fixing the covariance $\Sigma_{\theta }$ to a constant allows $\mu_{\theta }$ to be parameterized as a function of the noise, as defined in Equation 6:

$$\mu_{\theta }=\frac{1}{\sqrt{\alpha_{t}}}\cdot \left(x_{t}-\frac{1-\alpha_{t}}{\sqrt{1-{\bar{\alpha }}_{t}}}\cdot \epsilon_{\theta }\left(x_{t},t\right)\right).$$ (6)

This simplifies the objective function $L_{vlb}$ to a mean squared error loss over the noise prediction space, as defined in Equation 7:

$$L_{\text{simple }}=E_{t,x_{0},\epsilon }{\left\|\epsilon -\epsilon_{\theta }\left(x_{t},t\right)\right\|}^{2}$$ (7)

where $\epsilon_{\theta }$ is the noise predicted by the network. Once trained, this network enables ancestral sampling to recover clean data from random noise.

Score SDE (Song et al., 2021b) extends the discrete steps of DDPM into a continuous stochastic process described by a stochastic differential equation (SDE), allowing finer control over sample quality and diversity. Furthermore, it has been demonstrated that deterministic ordinary differential equations (ODEs), termed probability flow ODEs, can model the reverse process (Song et al., 2021b). By removing stochastic components, ODEs support larger integration step sizes and improve computational efficiency, enabling faster sampling frameworks such as PNDMs (Liu et al., 2022) and DPM-Solver (Lu et al., 2022b).

### 2.2 ConditionaL diffusion probabilistic models

To overcome the inherent randomness of unconditional DDPMs, conditional diffusion models introduce a conditioning variable c (such as class labels, text, or visual observations) to guide generation, formulated as $p_{\theta }\left(x|c\right)$ (Rombach et al., 2022). Guidance during sampling is primarily achieved via two paradigms: classifier guidance and classifier-free guidance.

*Classifier Guidance* uses a pre-trained external classifier to compute the gradient of the target class probability at each denoising step (Dhariwal and Nichol, 2021). This gradient modifies the noise prediction, steering the generation toward the desired condition.

*Classifier-free Guidance* eliminates the need for an external classifier by jointly training a single model on both unconditional and conditional score estimators, using a null token ∅ for the unconditional path (Ho and Salimans, 2022). This approach offers a superior trade-off between sample quality and diversity, providing a flexible and widely adopted mechanism for downstream robotic applications.

### 2.3 The recent development

Recent research has focused on overcoming the primary bottlenecks of traditional diffusion models, namely, high computational complexity, slow sampling speeds, and heavy data reliance.

#### 2.3.1 Diffusion process

Instead of operating a Wiener process directly in high-dimensional pixel space (Karras et al., 2022), Latent Diffusion Models (LDMs) perform the diffusion process within a compressed, low-dimensional latent space. Frameworks like LSGM (Vahdat et al., 2021) and INDM (Kim et al., 2021) utilize a VAE or a Normalizing Flow model to map inputs into a latent representation where the diffusion network is trained, significantly reducing computational overhead. Stable Diffusion (Rombach et al., 2022) leverages this architecture to incorporate cross-modal text conditions efficiently. Alternative forward processes have also been proposed to optimize trajectory dynamics, such as modeling trajectories as electric field lines (Xu et al., 2022), substituting Gaussian noise with deterministic degradations like blurring (Bansal et al., 2024), or employing optimized flow matching formulations (Lipman et al., 2023).

#### 2.3.2 Sampling acceleration

To reduce the high time complexity of multi-step iterative denoising, knowledge distillation transfers generative trajectories into few-step student models (Lopes et al., 2017). Progressive distillation (Salimans and Ho, 2022) recurrently halves sampling steps, while two-stage variants adapt this to conditional setups (Meng et al., 2023). Consistency models (Song et al., 2023) and related one-step methods (Luhman and Luhman, 2021; Berthelot et al., 2023) directly map noise to the data distribution in a single step, and Reflow (Liu X. et al., 2023) straightens sampling trajectories to achieve efficient one-step generation.

Alternatively, training-free methods accelerate sampling by utilizing advanced numerical solvers on pre-trained models. DPM-Solver (Lu et al., 2022b) utilizes analytical high-order ODE solutions to compress sampling steps, while EDM (Karras et al., 2022) implements a second-order Heun method to minimize discretization error. To optimize stochastic sampling, adaptive step sizes (Jolicoeur-Martineau et al., 2021) and restart mechanisms (Xu Y. et al., 2023) balance discretization errors against stochastic error correction.

#### 2.3.3 Likelihood optimization

While DDPM implicitly optimizes likelihood via the ELBO, exact maximum likelihood estimation remains challenging. ScoreFlow (Song et al., 2021a) couples normalizing flows with score matching to optimize log-likelihood, while Variational Diffusion Models (VDM) (Kingma et al., 2021; Huang et al., 2021) establish a continuous-time variational framework that directly optimizes likelihood bounds. Other approaches achieve tighter likelihood bounds by employing hybrid loss functions that explicitly reweight the variational terms (Nichol and Dhariwal, 2021; Lu et al., 2022a).

#### 2.3.4 Applications

Diffusion models have revolutionized image, video, and text generation. In robotics, conditional frameworks like Imagen (Saharia et al., 2022), Stable Diffusion (Rombach et al., 2022), and DALL-E 2 (Ramesh et al., 2022) provide critical multimodal synthesis capabilities, enabling semantic data scaling and visual prompt generation for task reasoning. Crucially, the capacity of diffusion models to capture highly non-linear, multimodal distributions allows them to directly model the joint space of visual observations and continuous actions. In this capacity, the diffusion model serves as a policy network, generating smooth, temporally consistent robot trajectories that avoid the averaging artifacts of classical policies, making them a foundational component in visuomotor control and motion planning.

## 3 Diffusion model in robotics

In this section, the application of diffusion models in various aspects of robot learning is discussed in detail, and the current state of development and future directions are analyzed.

To ensure the comprehensiveness and systematic rigor of this survey, our literature selection methodology was structured through a systematic scoping approach. We conducted a comprehensive electronic search across premier databases—including IEEE Xplore, ACM Digital Library, ScienceDirect, and Google Scholar—covering publications up to 2025. The search queries systematically targeted the intersection of generative physics and physical embodiments, structured as: (”diffusion models”) AND (”robotics” OR ”robot learning” OR ”trajectory planning”). Articles were rigorously included only if they presented peer-reviewed technical developments or top-tier conference proceedings focusing on deploying diffusion processes within continuous robotic state-action spaces, while pure image or video generation works lacking kinematic or visual-tactile feedback loops were systematically excluded. While our primary literature search prioritized peer-reviewed journal articles and top-tier conference papers, we explicitly expanded our inclusion criteria to encompass selected high-impact arXiv preprints. Given the exceptionally rapid evolution of diffusion models in robotics, relying exclusively on formally published literature would omit crucial state-of-the-art breakthroughs. To ensure academic rigor, these preprints were strictly screened based on their technical soundness, relevance, and origin from reputable research institutions.

Furthermore, to accurately reflect the real-time exponential growth and rapid iterative nature of this field, we additionally utilized the ArXiv preprint database specifically to analyze and visualize the overall publication trend, as presented in Figure 1.

### 3.1 Taxonomy of diffusion models in robotics

To systematically categorize the literature, this section introduces a structured, top-down taxonomic framework that serves as the organizational backbone of our survey. As conceptually illustrated in Figure 3, we establish a comprehensive hierarchical classification system structured around four primary domains that reflect the progressive evolution of embodied generative intelligence: environmental data preparation, decision-making control paradigms, high-level cognitive reasoning, and specialized mechanical or sensory applications.

**FIGURE 3:**
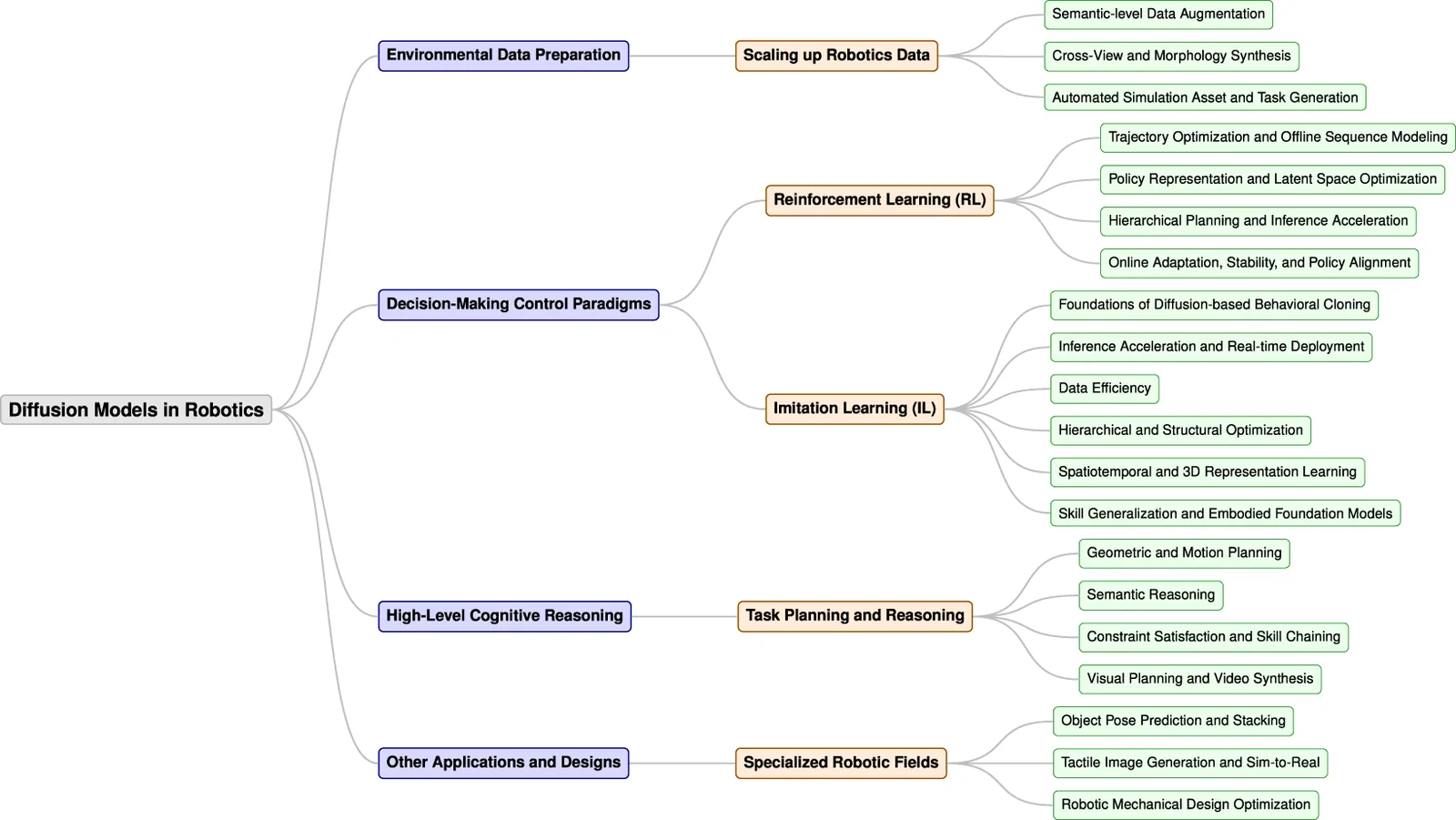
The proposed top-down hierarchical taxonomic framework of diffusion models in robot learning. The architecture systematically categorizes existing literature into four interconnected macro dimensions: environmental data preparation, decision-making control paradigms, high-level cognitive reasoning, and specialized robotic applications or designs.

At the foundational layer of the taxonomy, diffusion models function as generative “data factories” dedicated to Environmental Data Preparation. As detailed in Section 3.2, this paradigm leverages the generative priors of diffusion processes to address the critical data sparsity problem through systematic Scaling up Robotics Data. The literature under this category expands from asset-level semantic augmentation to cross-view scene synthesis and automated simulation asset generation, expanding the training distribution without manual collection overhead.

The core of our taxonomy classifies dynamic Decision-Making Control Paradigms along two complementary dimensions: robot learning paradigms and mathematical generation mechanisms.

- Reinforcement Learning (RL) Paradigm: Focuses on trial-and-error policy acquisition. Algorithmically, it is divided into macro-level Sequence Modeling (trajectory-centric optimization across long continuous time horizons), action-centric Policy Representation (latent space optimization for instantaneous outcomes), hierarchical planning frameworks, and the latest advancements in real-time Online Adaptation and stability alignment.
- Imitation Learning (IL) Paradigm: Focuses on learning directly from expert demonstrations. Mechanistically, it transitions from foundational behavioral cloning and inference acceleration to advanced structural optimizations, including data-efficient learning, 3D spatiotemporal representation learning, and large-scale embodied foundation models.

At the execution layer, diffusion models extend beyond low-level trajectory control to serve as agents for High-Level Cognitive Reasoning. Discussed under the umbrella of Task Planning and Reasoning, this paradigm treats high-level logical inference as a conditional denoising process. It decomposes abstract long-horizon missions into geometric motion plans, semantic subgoals, or video-based visual foresight to effectively bridge the execution gap in unstructured environments.

Finally, to ensure maximum taxonomic coverage, the framework encompasses Other Applications. This dimension categorizes expanding research frontiers that apply diffusion priors to Specialized Robotic Fields outside standard control loops, specifically detailing object pose prediction, tactile image generation for sim-to-real alignment, and generative robotic mechanical design optimization.

### 3.2 Scaling up robotics data

The field of AI has been booming in recent years. As a key force leading the future development of science and technology, it is changing our lives at an unprecedented speed. The development of AI cannot be separated from three aspects, algorithms, arithmetic, and data, which together drive the breakthroughs and innovations in AI technology. Countless scientific studies over the past decade have proven that large neural networks can produce remarkable results when combined with large datasets. Recent advancements in natural language processing (NLP) and computer vision (CV) have been primarily driven by the availability of large-scale, publicly accessible datasets collected from web sources.

In the field of robot learning, data sparsity is a key factor that limit progress. Many studies focus on algorithm innovation, demonstrating the robustness of algorithms only in limited environmental contexts, with a few objects to interact with and a small set of action skills. However, even state-of-the-art algorithms cannot achieve generalized embodied intelligence if a large amount of diverse data is missing. The desired data cover not only a wide range of motor skills, but also a wide range of different objects and visual domains. Several works (Jiang et al., 2022; Brohan et al., 2022) have demonstrated that using the same algorithms, robot learning algorithms perform more robustly and accurately when scaled to larger, more diverse datasets. When combined with large models of robot learning and large datasets, it is expected that higher performance robotic systems can be realized that can perceive, reason, and act in complex environments.

However, the acquisition of real-world robotics data is costly and time-consuming. The three usual methods for acquiring real-world robot data are kinesthetic teaching, teleoperation, and passive observation. All these methods require the involvement of experts and are cumbersome and time-costly. Moreover, making the training data cover a wide range of objects and scenes requires extensive physical resources. To address these issues, past work has typically expanded the data volume using simulated data, simple data augmentation, and domain randomization. While building simulated environments makes it easy to introduce different objects, backgrounds, and tasks, simulated environments differ from real environments in appearance and various parameters. And algorithms trained on these data have limited ability to generalize to the real world. Simple data augmentation scales up the data by cropping, flipping, color transformations, etc., but it essentially introduces no additional semantic information, and merely makes low-level changes to the appearance of the scene, which is insufficient for scene generalization. Domain randomization (Tobin et al., 2017; Mehta et al., 2020), on the other hand, varies the physical and rendering parameters in the simulation environment, but it is similarly constrained by the gap between the simulation and the real world.

The emergence of diffusion model opens up new ideas for the expansion of robotic data due to its powerful and realistic generation capabilities. As is shown in Figure 4, unlike traditional data augmentation methods, diffusion models achieve semantic-level data augmentation through novel asset generation. A systematic taxonomy of diffusion-based data scaling frameworks is provided in Table 1.

**FIGURE 4:**
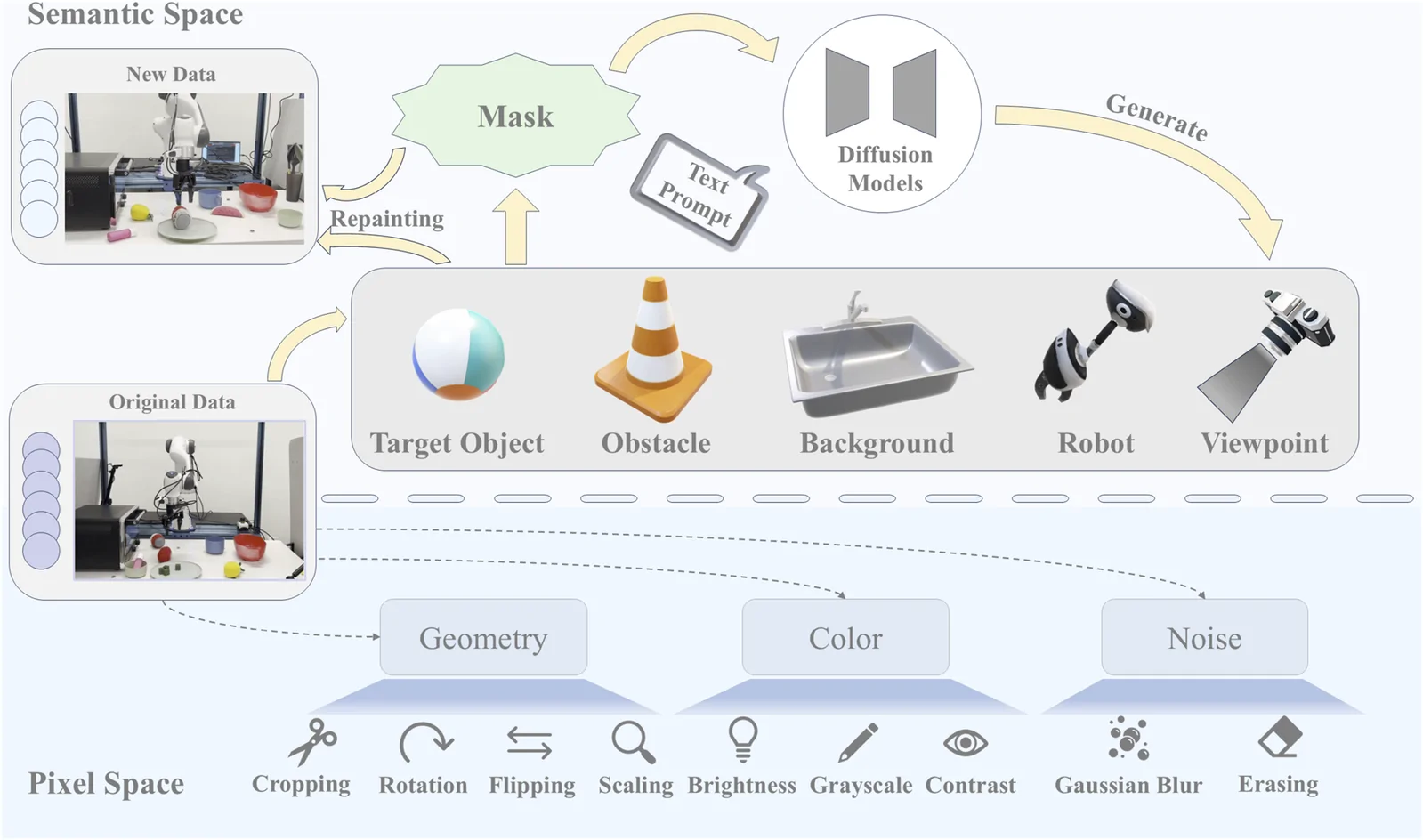
Paradigms of data augmentation in robot learning. The lower section classifies traditional pixel-space methods into geometric, color, and noise-based transformations. These techniques perform shallow modifications directly on image pixels to improve model robustness. In contrast, the upper section illustrates the generative workflow of diffusion models within the semantic space. By decoupling the scene into high-level variables such as target objects, obstacles, and camera viewpoints, diffusion models utilize text prompts and masks to synthesize diverse, physically plausible training samples. This approach enables the creation of complex environmental variations that go beyond simple pixel-level perturbations.

**TABLE 1:** Systematic taxonomy of diffusion-based robotic data scaling frameworks, contrasting target objectives, technical contributions, and experimental platforms to demonstrate the technological evolution from manual collection to automated synthesis.

Selected works	Year	Target problem	Contribution	Simulation platform and dataset
CACTI (Mandi et al., 2022)	2023	IL; Scaling up data	The first application of diffusion models for asset generation	CACTI-sim-100; Realworld kitchen
GenAug (Chen et al., 2023c)	2023	IL; Scaling up data	Refining generation details; combined generation of backgrounds objects, and distractors	LVIS; Real-world
ROSIE (Yu et al., 2023)	2023	IL; Scaling up data	Automating the generation process	RT-1
DMD (Zhang et al., 2024)	2024	IL; Data augmentation	Generating images with different object alignments and viewpoints	VIME; Realworld grabbing and pushing
RoVi-aug (Chen et al., 2024c)	2024	IL; Scaling up data	Generating new robots and new viewpoints	Robosuite simulator; Berkeley UR5
SYNTHER (Lu et al., 2024)	2024	RL; Scaling up data	Combining experience replay with diffusion models to expand the experience data	D4RL; DMC; OpenAI gym; V-D4RL
Gen2Sim (Katara et al., 2024)	2024	RL; Scaling up data	Automating the generation of simulation environments, tasks, and reward functions	IsaacGym and Twin environment in reality
RoboGen (Wang et al., 2024d)	2024	RL; Scaling up data	Decomposing tasks and selecting the best learning method using a large language model	Genesis

#### 3.2.1 Semantic-level data augmentation

Unlike traditional pixel-level transformations, semantic-level data augmentation leverages the generative priors of diffusion models to synthesize novel, physically plausible scene elements while preserving the underlying task logic. This paradigm allows for the diversification of environmental factors, such as asset appearances, textures, and background contexts that are crucial for out-of-distribution (OOD) generalization.

CACTI (Mandi et al., 2022) uses diffusion models for the first time to generate offline datasets for robot imitation learning. CACTI (Mandi et al., 2022) replays expert or agent demonstrations multiple times, with each replay involving manual changes to the layout of some obstacles in the scene, ensuring the data is sufficiently diverse. Next, a manual mask is applied to the obstacles that need to be replaced. The mask guarantees the stability of generation, leaving portions of the scene outside the mask unaltered, and stable diffusion is used to generate new objects, along with a text prompt pertaining to the created object. Experimental results show that CACTI enables a single policy to achieve a 62% success rate across 18 semantic tasks and 100 layout variations in simulation. Specifically, the proposed semantic in-painting augmentation provides a performance gain of 15%–20% in absolute success rate compared to variants using only standard image augmentations. However this work is only applied in a kitchen environment and generation is limited to task-independent objects. This highlights an initial trade-off: while manual masking ensures generation stability, it inherently limits scalability and functional diversity.

GenAug (Chen et al., 2023c) instead generates obstacles, task objects, and backgrounds. The method still involves masking the objects to be replaced, providing a text prompt, and using a text-to-image diffusion model for generation. However, GenAug (Chen Z. et al., 2023) addresses some details in the generation process, such as the fact that cross-category generation might significantly alter the shape and size of objects, which could overlap with the robot’s original trajectory. Therefore, this work first renders an object of the same size from a mesh dataset, without rendering visual details, and then uses a depth-aware diffusion model to generate the complete object. After generation, it checks whether the bounding boxes of obstacles and task objects overlap and discards the overlapped ones. Compared to CACTI, GenAug advances the synthesis pipeline by explicitly incorporating 3D geometric constraints to prevent physically impossible scenarios, though it still relies on a human-in-the-loop masking process.

ROSIE (Yu et al., 2023) uses a target detection model with a segmentation head to automatically perform object detection and segmentation. It further utilizes a large language model to refine and generate the text prompts needed for data augmentation. This work employs Imagen (Saharia et al., 2022) to generate new objects and backgrounds and thus has automated the generation process. By replacing the manual interventions seen in CACTI and GenAug with automated segmentation and LLM prompting, ROSIE resolves the primary scalability bottleneck in semantic data synthesis.

DMD (Zhang et al., 2024) enhances algorithm robustness in in-distribution scenarios by employing diffusion models to generate images with slight variations in object arrangements and viewing angles within identical scenes. These synthesized images are subsequently utilized for imitation learning to train the policy. The proposed method demonstrates improved success rates in both object grasping and pushing tasks compared to conventional approaches. Unlike the aforementioned methods that prioritize out-of-distribution semantic diversity, DMD’s critical insight is that minor geometric perturbations are often more crucial for achieving control stability during physical deployment.

#### 3.2.2 Cross-view and morphology synthesis

Beyond modifying environmental assets, another dimension of data scaling involves diversifying the embodiment parameters, such as camera configurations and robot morphologies. In real-world deployments, robots often encounter viewpoints or structural variations not present in the training demonstrations. To address this, recent works have leveraged 3D-aware diffusion models and multi-stage generative pipelines to synthesize data.

RoVi-Aug (Chen et al., 2024c) introduces a new generation approach by creating different robots and viewpoints, thereby generalizing the algorithm to different robots and new camera perspectives. For robot generation, the work segments the robot in the image using the Segment Anything Model (Kirillov et al., 2023) and applies a mask. A fine-tuned stable diffusion model (Rombach et al., 2022) is used to generate the robot image, and the E2FGVI (Li et al., 2022) inpainting model fill the masked area with suitable images. For viewpoint generation, a 3D perception diffusion model, ZeroNVS (Sargent et al., 2024), is used to generate images from different viewpoints. This dual-track augmentation enables imitation learning policies to generalize across different hardware platforms and camera setups without requiring additional real-world collection.

This category of augmentation is particularly significant for Embodied AI, as it explicitly decouples the learned manipulation skills from specific hardware constraints, paving the way for more universal and platform-agnostic robotic policies.

#### 3.2.3 Automated simulation asset and task generation

Beyond real-world visual augmentation, diffusion models are increasingly leveraged to bridge the gap between abstract task descriptions and high-fidelity simulation environments. By integrating with Large Language Models (LLMs), diffusion models can automate the creation of 3D assets and scene layouts, enabling the scalable generation of diverse training environments for RL and motion planning.

SYNTHER (Lu et al., 2024) combines experience replay with diffusion models to flexibly expand the experience data collected by agents. In offline settings, SYNTHER uses diffusion models to augment small offline datasets, and this additional synthetic data can effectively train more complex networks. In online reinforcement learning, the additional data allows agents to use much higher update-to-data ratios, leading to increased sample efficiency.

Moving towards full automation, Gen2Sim (Katara et al., 2024) and RoboGen (Wang et al., 2024d) combine the logical reasoning of GPT-4 with the generative power of diffusion models. Gen2Sim represents a significant step toward this goal by scaling up robotic manipulation data through “web-to-simulation” transfer. The process begins with GPT-4 (OpenAI, 2023) generating a taxonomy of task-relevant objects. To ground these concepts in 3D space, the framework utilizes Stable Diffusion (Rombach et al., 2022) to retrieve or generate high-quality 2D images, which are then lifted into 3D meshes using Zero-1-to-3 (Liu R. et al., 2023). This 3D-aware diffusion model ensures multiview consistency during the reconstruction of object geometries.

RoboGen (Wang et al., 2024d) further advances this autonomy by establishing a self-evolving framework that automates the complete cycle of robotic skill acquisition. Rather than focusing exclusively on asset synthesis, the system utilizes LLMs to partition complex, high-level missions into a series of structured sub-tasks. For each component, the framework autonomously determines the optimal learning strategy, such as reinforcement learning, motion planning, or trajectory optimization. Within this pipeline, diffusion models serve a dual purpose: synthesizing diverse 3D simulation environments and generating the necessary training objectives.

Although the above work greatly scales up the robot learning data, there are still some problems in practical applications. First, whether using text-to-image or image-to-image diffusion models, they only generate objects, backgrounds, viewpoints, and so on, without generating new robot actions or trajectories. Essentially, they do not help the robot learn new skills. Second, since the generation process involves multiple tasks, such as segmentation, generation, inpainting, and policy training, the cascading of multiple models may lead to error propagation, affecting the final result. Additionally, the generation speed is relatively slow. Lastly, because images are generated frame by frame, there is some loss of temporal consistency between frames, causing small visual differences between them. Future work may focus on solving these issues.

### 3.3 Diffusion model in reinforcement learning

Reinforcement learning (RL) is one of the most commonly used methods in robot learning, where an agent learns the optimal policy through interaction with the environment by maximizing the reward function. Despite its widespread use in robotics, RL still faces several challenges. First, the application of online RL is limited because it requires a large amount of interaction data to learn effective strategies. However, in the real world, robot exploration and task attempts can be very expensive and time-consuming. While offline RL mitigates the issue of low sample efficiency, it remains constrained by the limited coverage of static datasets. In addition, rewards are often delayed or sparse in robotic tasks, meaning the robot will not receive timely feedback during the learning process. In complex robot tasks, the dimensions of the state space and action space are particularly large, which not only increases the difficulty of robot learning but also leads to high computational complexity and memory consumption.

In recent years, a series of works has integrated diffusion models into the RL framework with notable success. The inherent suitability of diffusion models for RL stems from their exceptional capacity to represent complex, multimodal action distributions, which often baffle traditional Gaussian-based policies. Furthermore, by framing decision-making as a global trajectory denoising process, diffusion models inherently maintain temporal coherence across long horizons. Their flexible conditioning mechanism also allows for the seamless integration of task-specific constraints and rewards without the need for extensive retraining, thereby addressing the long-standing challenges of data scarcity and high-dimensional optimization in robotic learning. A categorization of diffusion models in reinforcement learning is provided in Table 2.

**TABLE 2:** A categorization of diffusion models in reinforcement learning across various paradigms to highlight technical innovations in policy expressiveness and hierarchical trajectory optimization within the current robotic research landscape.

Selected works	Year	Target problem	Contribution	Simulation platform and dataset
Diffuser (Janner et al., 2022)	2022	Model-based RL	Pioneering the application of diffusion models to trajectory optimization in reinforcement learning	D4RL; Block stacking
Diffusion-QL (Wang et al., 2023b)	2023	Offline RL	Integrating diffusion models with offline reinforcement learning, employing Q-value guidance to steer the diffusion learning	D4RL
SfBC (Chen et al., 2023a)	2023	Offline RL	Decoupling a strategy into an expressive generative behavior model and an action evaluation model	D4RL
DiffCPS (He et al., 2023)	2023	Offline RL	Developing a primal-dual method to address constrained policy search in diffusion models	D4RL
SRDP (Ada et al., 2024)	2024	Offline RL	Employing state reconstruction signals to guide diffusion policies, mitigating distribution shift caused by out-of-distribution states	D4RL
EDGI (Brehmer et al., 2024)	2024	Model-based RL	Introducing an equivariant diffusion model that improves sample efficiency	3D navigation; Block stacking
IDQL (Hansen-Estruch et al., 2023)	2023	Implicit Q-Learning	Replacing implicit Gaussian policies with diffusion-based policies, strengthening implicit Q-learning performance	D4RL
Decision diffuser (Ajay et al., 2023a)	2023	Offline RL	Employing classifier-free guidance with low-temperature sampling, applying inverse dynamics models to derive decision actions	D4RL
Energy-guided Diffusion Sampling (Lu et al., 2023)	2023	Offline RL	Proposing a new training objective called comparative energy prediction to guide the generation process of diffusion models	D4RL
LDCQ (Venkatraman et al., 2024)	2024	Offline RL	Integrating latent diffusion models with offline reinforcement learning, reducing suboptimal trajectories and extrapolation errors	D4RL
MetaDiffuser (Ni et al., 2023)	2023	Offline Meta-RL	Introducing diffusion models as conditional planners to enhance offline Meta-RL performance and generalization capability	2D navigation; Multi-task MuJoCo
AdaptDiffuser (Liang et al., 2023)	2023	Offline RL	Enhancing task generalization capability through adaptive synthetic data generation and diffusion model fine-tuning	D4RL; KUKA robot
DiffuserLite (Dong et al., 2024)	2024	Offline RL	Refining trajectory generation through coarse-to-fine planning to reduce the search space	D4RL; FinRL; Robomimic
HDMI (Li et al., 2023a)	2023	Offline RL; Hierarchical	Deploying a cascade framework where the goal diffuser discovers sub-goals and then synthesizes corresponding action sequences	D4RL
Hierarchical Diffuser (Chen et al., 2024a)	2024	Offline RL; Hierarchical	Implementing hierarchical planning with a high-level sparse diffuser and a low-level trajectory generator	D4RL
MADiff (Zhu et al., 2023)	2023	Offline RL; Multi-agent	Introducing diffusion models to multi-agent offline reinforcement learning tasks for the first time	MPE; MA mujoco; SMAC; MATP
DOM2 (Li et al., 2023b)	2023	Offline RL; Multi-agent	Enhancing policy performance by expanding the dataset through replication of trajectories exceeding return thresholds	MPE; MA MuJoCo
DIPO (Yang et al., 2023b)	2023	Online RL	Pioneering the integration of diffusion models into online reinforcement learning	MuJoCo
CPQL (Chen et al., 2024d)	2024	Online RL	Introducing a consistency policy framework that establishes a one-step mapping from noise to target policy	D4RL; dm_control
PolyGRAD (Rigter et al., 2024)	2024	Online RL	Proposing the first autoregressive world model framework capable of generating full trajectories	MuJoCo
DACER (Wang et al., 2024c)	2024	Online RL	Formulating the reverse process of diffusion models as policy functions, using Gaussian mixture models to fit policy distributions	MuJoCo
DPD (Zhang et al., 2025b)	2025	Offline RL	Compressing multi-step diffusion models into single-step deterministic policies via knowledge distillation	D4RL
GenPO (Ding et al., 2025)	2025	Online RL	Resolving exact log-likelihood computation via dual action spaces to integrate diffusion models with on-policy RL	MuJoCo
RSM (Ma et al., 2025)	2025	Online RL	Introducing a reweighted score matching objective to bypass optimal policy sampling for highly efficient online RL	MuJoCo
OTPR (Sun et al., 2025)	2025	Online RL	Utilizing optimal transport and Q-functions to stabilize the online fine-tuning of diffusion-based imitation policies	Robomimic; CALVIN; Franka-kitchen
Multi-loco (Yang et al., 2025)	2025	Online RL; Legged multi-robot	Unifying diverse legged robots via a morphology-agnostic diffusion prior and a shared residual RL policy to bridge the sim-to-real gap	Quadrupeds; bipeds; Real-world
Chaos-RL (Zhang et al., 2025a)	2026	Online RL; Legged robustness	Enabling critics to evaluate multiple valid action options and penalizing shaky movements to keep legged robots from falling	MuJoCo; TonyPi robot
DreamControl (Kalaria et al., 2025)	2025	Online RL; Loco-manipulation	Using a human-motion diffusion prior to guide RL, solving the exploration bottleneck for complex whole-body humanoid tasks	Unitree G1; IsaacLab

#### 3.3.1 Trajectory optimization and offline sequence modeling

The integration of diffusion models into offline RL has fundamentally redefined trajectory optimization by shifting the focus from localized Markovian transitions to global sequence synthesis. Diffuser (Janner et al., 2022) represents the pioneering work in this domain, framing planning as a non-autoregressive denoising process. Unlike conventional model-based approaches that often suffer from compounding errors in recursive prediction, Diffuser operates across localized receptive fields to ensure cumulative temporal consistency throughout the entire planning horizon. This holistic planning is particularly advantageous for complex sequential tasks such as long-horizon block stacking and pick-and-place, where temporal coherence is critical for task success. Building upon this, EDGI (Brehmer et al., 2024) incorporates spatial, temporal, and permutation symmetries into an equivariant diffusion architecture. This advancement directly addresses the data intensity limitations of generative models, enabling more robust autonomous navigation by leveraging the inherent geometric symmetries of unstructured environments.

A critical advancement in trajectory steering involves the ability to guide the denoising process toward high-performance outcomes using task-specific objectives. Decision Diffuser (Ajay et al., 2023a) utilizes a conditional generative model for sequential decision-making that employs classifier-free guidance and low-temperature sampling. By conditioning the generative process on high-reward signals, the framework captures the best decision-making actions within a dataset while utilizing an inverse dynamics model to extract executable actions from the synthesized state sequences. To ensure that these generated plans remain within the support of the offline training distribution, DiffCPS (He et al., 2023) introduces a primal-dual framework that establishes strong duality conditions for constrained policy search. Addressing a similar challenge of distribution shift, SRDP (Ada et al., 2024) employs state reconstruction signals to guide diffusion policies, effectively mitigating the extrapolation errors caused by out-of-distribution (OOD) states. Similarly, Energy-Guided Diffusion Sampling (Lu et al., 2023) utilizes a non-standardized energy function and a comparative energy prediction objective to provide precise intermediate guidance signals. This ensures convergence toward high-value regions even in complex robot control tasks where rewards are sparse or inconsistent.

Furthermore, addressing the challenge of generalization to unseen tasks has led to the development of Offline Meta-Reinforcement Learning architectures. MetaDiffuser (Ni et al., 2023) utilizes a pre-trained context encoder to project task-relevant information into a conditional diffusion manifold, enhancing the kinetic consistency of generated trajectories. This meta-adaptation capability is essential for multi-task learning scenarios, where agents must adapt to varied environmental dynamics. Complementing this, AdaptDiffuser (Liang et al., 2023) introduces a self-evolutionary mechanism that generates rich, composite expert data using reward gradients as guidance. By fine-tuning the model on high-quality synthetic data, AdaptDiffuser demonstrates superior performance and significantly improves success rates in physical deployments, specifically for continuous control tasks on a real-world KUKA robotic arm without necessitating additional real-world expert demonstrations. These trajectory-centric paradigms establish a unified foundation for long-horizon reasoning and robust offline adaptation.

#### 3.3.2 Policy representation and latent space optimization

While sequence-level planning treats RL as a holistic generative task, another pivotal line of research focuses on re-imagining the policy function itself as a conditional diffusion process. Traditional RL policies often struggle with multimodal expert data where multiple valid strategies exist for the same state, typically resulting in suboptimal “averaged” behaviors when using Gaussian priors. Diffusion-based policies resolve this by decoupling expressiveness from simple parametric forms, allowing the agent to learn a highly flexible action prior that can represent arbitrary, sharp distributions. Diffusion-QL (Wang Z. et al., 2023) exemplifies this by splitting the optimization objective into a behavioral cloning term and a Q-learning term, thereby ensuring that the sampled actions remain within the behavioral manifold of the training dataset while maximizing high-value outcomes.

To enhance the representational capacity further, frameworks such as SfBC (Chen H. et al., 2023) decouple policy learning into an expressive generative behavior model and a separate action evaluation critic. This structural separation allows the target distribution to be modeled with greater fidelity, effectively mitigating performance degradation in the presence of conflicting expert trajectories. Extending this decoupled architecture to multi-embodiment domains, Multi-Loco (Yang et al., 2025) introduces a unified framework that combines a morphology-agnostic diffusion prior with a lightweight residual RL policy. While this hybrid representation effectively bridges the sim-to-real gap across varied platforms by isolating shared kinematic patterns, its reliance on zero-padding limits its scalability to radically different morphologies, suggesting that future representations must natively encode structural topology. Similarly, IDQL (Hansen-Estruch et al., 2023) demonstrates that by compressing inputs into a low-dimensional latent space, the model can significantly reduce computational overhead and extrapolation errors in Q-learning. This dimensionality reduction establishes a tractable framework for mastering intricate robotic control tasks that were previously computationally prohibitive.

#### 3.3.3 Hierarchical planning and inference acceleration

Despite the expressive power of diffusion-based RL, the iterative nature of the denoising process introduces a severe inference bottleneck, with frequencies often falling below the requirements for real-time control. To resolve this, researchers have focused on hierarchical planning and sampling acceleration techniques. DiffuserLite (Dong et al., 2024) and Hierarchical Diffuser (Chen C. et al., 2024) employ a coarse-to-fine refinement strategy where a high-level sparse planner identifies distant subgoals, effectively reducing the planning search space. A low-level goal-conditioned policy then generates specific trajectories between these waypoints. This hierarchical approach is particularly suitable for long-horizon simulated tasks, enabling robots to maintain a larger receptive field and lower computational cost. Furthermore, HDMI (Li W. et al., 2023) utilizes a cascaded framework where a Transformer-based trajectory diffuser captures complex dependencies between subgoals more effectively than traditional U-Net structures, ensuring the stability of long-term plans in complex environments like Franka-Kitchen.

To achieve the millisecond-level feedback required for dynamic hardware, knowledge distillation has emerged as a vital acceleration paradigm. DPD (Zhang J. et al., 2025) introduces a framework that compresses multi-step diffusion teacher models into deterministic student policies that require only a single iteration for execution. By matching the student’s output directly with the target action distribution of the teacher, this method substantially increases inference speed without compromising the original reward performance. This acceleration is pivotal for autonomous systems that require rapid reactivity to unexpected state perturbations. Complementing these algorithmic shifts, structural optimizations in multi-agent systems have also gained traction. MADiff (Zhu et al., 2023) utilizes an innovative attention-based architecture to enable information exchange across latent layers of multiple agents, facilitating effective coordination in shared generative manifolds. Similarly, DOM2 (Li Z. et al., 2023) enhances policy performance by replicating high-reward trajectories within the dataset, thereby increasing learning efficiency and preventing agents from becoming trapped in local optima during joint-level control of collaborative autonomous swarms.

#### 3.3.4 Online adaptation, stability, and policy alignment

The frontier of robotic RL involves transitioning from static offline priors to dynamic online adaptation, where agents must refine their strategies through real-time interaction. While early works like DIPO (Yang L. et al., 2023) and CPQL (Chen Y. et al., 2024) introduced action relabeling and consistency policies to achieve faster updates, they were frequently constrained by the mathematical intractability of calculating exact action log-likelihoods for gradient updates. Recent breakthroughs in 2025 have addressed these theoretical barriers. GenPO (Ding et al., 2025) introduces a dual action space mechanism that allows for the precise computation of these log-likelihoods, successfully integrating diffusion models with stable on-policy techniques such as Proximal Policy Optimization. This contribution facilitates highly stable policy learning during continuous interaction with the environment, addressing a major limitation in the online exploration capabilities of simulated agents in dynamic continuous control environments. However, standard on-policy actor-critic frameworks often struggle with the dynamic instability inherent to legged robots. To tackle this, (Zhang H. et al., 2025) introduce a chaos-augmented RL framework that employs Kernel Density Expected Advantage Estimation to evaluate multimodal diffusion outputs, alongside Maximum Lyapunov Exponent regularization. While this significantly suppresses chaotic divergence during online interaction, estimating local state divergence through experience replay makes the policy’s stability highly sensitive to buffer density and sampling quality. Advancing beyond pure locomotion, DreamControl (Kalaria et al., 2025) tackles the formidable exploration bottleneck of whole-body humanoid loco-manipulation. By leveraging a human-motion diffusion prior to generate kinematic reference trajectories, it provides explicit guidance for the RL policy. This human-inspired prior allows the humanoid to reliably discover complex, coordinated solutions—such as bracing to open heavy drawers or squatting to lift boxes—that remain entirely unattainable through direct, unguided RL exploration.

Furthermore, the research community is exploring reweighted score matching and optimal transport to stabilize the online fine-tuning of diffusion policies. RSM (Ma et al., 2025) bypasses the need for optimal target samples by generalizing the standard score matching objective, while OTPR (Sun et al., 2025) treats the policy as an optimal transport map, utilizing the Q-function as a transport cost to maintain stability. Notably, OTPR has demonstrated robust performance in real-world environments like Franka-Kitchen, where aligning policy outputs with geometric evolution ensures stable and robust fine-tuning despite distribution shifts. Advancing toward broader environmental awareness, PolyGRAD (Rigter et al., 2024) and DACER (Wang et al., 2024c) introduce non-autoregressive world models and Gaussian mixture modeling to regulate the balance between exploration and exploitation. Despite these efforts, achieving millisecond-level feedback in dynamic online loops remains a non-trivial challenge. Future research must prioritize hardware-efficient, safety-constrained diffusion frameworks that can handle the sensor noise, actuator backlash, and unpredictable friction characteristic of real-world robotic deployment in physical environments.

In summary, the transition from “clean” simulations to unstructured physical reality requires a fundamental shift toward safety-constrained and computationally sustainable diffusion frameworks. The research discussed above highlights a paradigm shift where diffusion models serve as both holistic planners and high-capacity policy approximators. By bridging the gap between high-level reasoning and low-level physical control, these generative models provide a robust cognitive architecture for the next-generation of autonomous robotic systems.

### 3.4 Diffusion model in imitation learning

Imitation Learning (IL), particularly through Behavioral Cloning (BC), has emerged as a powerful paradigm for robotic skill acquisition, allowing agents to map complex visual observations directly to actions by leveraging expert demonstrations. Unlike RL, which often suffers from high sample complexity and the “reward engineering” challenge, IL is particularly suited for tasks where the reward signal is sparse or the optimal behavior is intuitive for humans but difficult to mathematically define, such as cloth folding or surgical assistance.

Traditional IL often fails when expert demonstrations contain multiple valid strategies because standard Gaussian-based models tend to average these different behaviors. Diffusion models address this by treating policy learning as conditional density estimation, which allows them to capture the full distribution of expert actions. Instead of converging on a single average, these models represent the complete manifold of behaviors so the robot can follow distinct expert modes accurately. In the context of visuomotor control, diffusion models map high-dimensional visual observations to continuous actions by generating entire sequences. This method reduces the drift caused by execution errors and ensures the resulting movements are smooth and consistent with the expert goals. A systematic categorization of diffusion-based imitation learning studies is provided in Table 3.

**TABLE 3:** A systematic categorization of diffusion-based imitation learning research comparing target problems and technical contributions across diverse platforms to illustrate the progression from basic behavioral cloning to advanced 3D-aware and foundation-scale robotic policies.

Selected works	Year	Target problem	Contribution	Simulation platform and dataset
(Pearce et al., 2023)	2023	BC	Pioneering the application of diffusion models to BC, demonstrating effectiveness in imitating human behaviors	D4RL; kitchen Environment
BESO (Reuss et al., 2023)	2023	IL	Utilizing score-based diffusion models for action generation for the first time	CALVIN; block-push; Relay kitchen
ChainedDiffuser (Xian et al., 2023)	2023	IL; Hierarchical	Applying diffusion models to a hierarchical framework for imitation learning to generate trajectories	RLBench; Real-world
Diffusion Co-policy (Ng et al., 2023)	2023	IL	Proposing a transformer-based diffusion model to predict joint action sequences in human-robot collaborative tasks	Motion planning in Simulation and real-world
Diffusion Policy (Chi et al., 2023)	2023	IL	Formulating robotic visuomotor policies as conditional denoising diffusion processes to generate robot actions	Robomimic; Push-T; Multimodal block pushing; Franka kitchen; real-world
Consistency Policy (Prasad et al., 2024)	2024	IL	Introducing consistency distillation that transfers teacher model knowledge to the student policy	Robomimic; Push-T; Franka kitchen; real-world
VDD (Zhou et al., 2024a)	2024	IL	Compressing a diffusion model into a mixture-of-experts framework accelerating inference speed	Relay kitchen; block-push; D3IL
SDP (Høeg et al., 2024)	2024	IL	Proposing an action buffering mechanism that implements partial denoising trajectories, accelerating inference speed	Robomimic; Push-T; Real-world Push-T
BRIDGER (Chen et al., 2024b)	2024	IL	Optimizing reverse process initialization that replaces Gaussian noise with the source policy	Franka kitchen; Adroit; 6-DoF grasp; real-world
Equivariant Diffusion policy (Wang et al., 2024b)	2024	IL	Incorporating equivariance into diffusion policy design, utilizing domain symmetry to boost training sample efficiency	MimicGen; Real-world
MDT (Reuss and Lioutikov, 2023)	2023	IL; Foundation	Combining multimodal transformers with pretrained foundation models to learn long-horizon manipulation tasks	CALVIN
Crossway Diffusion (Li et al., 2024)	2024	IL	Incorporating a self-supervised auxiliary objective alongside the standard diffusion loss to optimize the model	Robomimic; Push-T; real-world
HDP (Ma et al., 2024)	2024	IL; Hierarchical	Integrating diffusion policy into a hierarchical framework, leveraging differentiable dynamics to optimize action generation	RLBench; Real-world
DISCO (Hao et al., 2024)	2024	IL; Hierarchical	Using VLMs to generate keyframes which serve as coarse guidance in a diffusion model for robotic motion generation	CALVIN; block-push; Franka kitchen; Push-T; Grasp pose; real-world
Track2Act (Bharadhwaj et al., 2024)	2024	IL; Hierarchical	Predicting plausible high-level plans using internet videos and enabling efficient fine-tuning with minimal robot data	Real-world
DP3 (Ze et al., 2024)	2024	IL; 3D	Presenting the first work integrating 3D visual representations into diffusion policy	MuJoCo; sapien; IsaacGym; PlasticineLab; real-world
3D diffuser Actor (Ke et al., 2024)	2024	IL; 3D	Encoding and concatenating 3D scene representations with text instructions and robot proprioceptive states	RLBench; CALVIN; real-world
DNAct (Yan et al., 2024)	2024	IL; 3D	Using NeRF to pretrain 3D encoders, distilling 2D semantic features into 3D space via neural rendering	RLBench; Real-world
R&D (Vosylius et al., 2024)	2024	IL	Unifying robotic motions and RGB observations in a shared image space, making action align through diffusion models	RLBench; Real-world
ISS policy (Xia et al., 2025)	2025	IL; Spatiotemporal	Introducing an implicit scene supervision mechanism to align policy outputs with geometric evolution	Adroit; MetaWorld; Real-world
DP4 (Liu et al., 2025)	2025	IL; Spatiotemporal	Constructing a dynamic four-dimensional Gaussian world model to grant precise spatiotemporal anticipation capabilities	Adroit; DexArt; RLBench; real-world
DisDP (Vanjani et al., 2025)	2025	IL; Robustness	Proposing a framework based on disentangled representations to maintain stability against sensor noise and occlusions	The colosseum; Libero
NoMaD (Sridhar et al., 2024)	2024	IL; Navigation	Generating action sequences for navigation tasks using diffusion policy, achieving exploration and navigation mode switching	6 distinct indoor and Outdoor environments
(Yoneda et al., 2023b)	2023	IL	Ensuring fidelity of human inputs and congruence maintenance of target behaviors in human-robot collaborative control	D4RL; open AI gym; Lunar reacher; block pushing
RDT (Liu et al., 2024)	2024	IL; Foundation	Proposing the first diffusion foundation model for dual-arm robotic manipulation that overcomes the data heterogeneity problem	46 datasets of Various robots for pretraining; 7 tasks for test
Xskill (Xu et al., 2023a)	2023	IL	Proposing a skill-conditioned policy to acquire skills from unlabeled human demonstrations	Franka kitchen; Real-world kitchen
Flow guided (Jung et al., 2025)	2025	IL; Acceleration	Replacing standard denoising with a flow matching architecture to overcome latency and open-loop execution limits	Push-T; MimicGen; Real-world
FDPP (Chen et al., 2025b)	2025	IL; Alignment	Incorporating human preference feedback to continuously fine-tune diffusion policies for dynamic intent alignment	Push-T; MimicGen
DiffuseLoco (Huang et al., 2025)	2025	IL; Acceleration	Optimizing diffusion models for 100 Hz torque-level inference to enable real-time multimodal legged locomotion	Quadrupeds; Bipeds
(He et al., 2024)	2024	IL; Hierarchical	Introducing a hierarchical diffusion framework to seamlessly coordinate end-effector manipulation with quadrupedal legged mobility	Quadrupeds; Real-world

#### 3.4.1 Foundations of diffusion-based behavioral cloning

One of the major advantages of diffusion models in IL is their ability to capture multimodal action distributions. As demonstrated by several works (Hegde et al., 2023; Chen L. et al., 2023), the flexible conditioning mechanisms allow these models to select and combine distinct behaviors, and even generate policies for specific maneuvers through textual descriptions. Early work on integrating diffusion models into imitation learning pivoted toward isolating and modeling the stochastic nuances of expert behavior in sequential tasks.

Pearce et al. (2023) were the first to apply diffusion models to behavior cloning for imitating human behavior in sequential environments. This work demonstrates the ability of diffusion models to model complex action distributions in robot control tasks and game environments. However, this work also highlights fundamental bottlenecks, such as increased inference time and numerous hyperparameters. Meanwhile, BESO (Reuss et al., 2023) uses score diffusion models to generate actions. It decouples the score model from the inference sampling process, allowing for faster sampling of policies and significantly reducing the number of denoising steps. Similarly, ChainedDiffuser (Xian et al., 2023) learns policies from demonstrations by combining high-level macroscopic action prediction with low-level trajectory diffusion generation, achieving satisfactory efficacy even with limited demonstration data and monocular views. Diffusion Co-policy (Ng et al., 2023) employs a Transformer-based diffusion model to predict joint action sequences of humans and robots in collaborative tasks. By conditioning on historical human actions, the robot can better adapt to human behavior while also adjusting to changing environments.

Early studies applied diffusion models to behavioral cloning, yet their reliance on single-step action generation limited their effectiveness in complex real-world tasks. Diffusion Policy (Chi et al., 2023) addresses this by extending the denoising process from isolated actions to trajectory-level generation. By integrating visual conditioning, this framework enables end-to-end imitation learning for robotic systems. It represents visuomotor policies as a conditional denoising process, employing a Transformer-based backbone to mitigate the over-smoothing effects often found in CNN-based models. The use of receding horizon control further allows for high-frequency action updates and precise speed control. These innovations enable the model to capture multimodal distributions and predict future action sequences while maintaining training stability. The operational robustness of this framework is evidenced by its successful deployment on physical Franka Emika Panda robotic arms for tasks such as precise Push-T maneuvers and multimodal object interaction, confirming its effectiveness in real-world robotic manipulation. Despite these strengths, the approach remains dependent on demonstration quality and faces challenges related to computational cost and inference latency, which subsequent research has sought to resolve.

#### 3.4.2 Inference acceleration and real-time deployment

Consistency Policy (Prasad et al., 2024) is engineered to mitigate the inference speed bottleneck of Diffusion Policy (Chi et al., 2023), enabling it to perform robot control tasks more efficiently. This work uses consistency distillation to transfer knowledge from a teacher model to a student model, enabling faster action generation in just one or three inference steps. VDD (Zhou H. et al., 2024) also uses a distillation approach to distill the diffusion model into a mixture of experts. VDD (Zhou H. et al., 2024) optimizes each expert by pre-training the diffusion model with a score function, which enables knowledge transfer between models through variational inference. This approach combines the strong generative capabilities of diffusion models with the efficient inference and interpretability of a mixture of experts. Although the distillation technique reduces the number of steps required for action prediction, this trade-off occasionally compromises sample structural diversity and visual fidelity.

To mitigate potential compromises to sample integrity, SDP (Høeg et al., 2024) introduces an action buffer that generates partially denoised action trajectories, where the immediate action is noise-free, and the noise level gradually increases for subsequent actions. By recursively updating this buffer, SDP enables recursive sampling. This approach improves inference speed while maintaining performance on par with traditional Diffusion Policy. This recursive rolling mechanism has been validated in real-world Push-T tasks, where it enables the robot to maintain interactive frequencies and robustly respond to dynamic perturbations. BRIDGER (Chen K. et al., 2024) accelerates inference speed by optimizing the initialization of the reverse diffusion process, resembling a warm-start approach. It creates a connection between the source policy and the target policy through random interpolation. The source policy, which can be a task-based heuristic or a data-driven policy, serves as the starting point of the diffusion process instead of the conventional Gaussian noise initialization.

While the aforementioned methods focus on optimizing the standard diffusion process, recent research explores alternative generative frameworks to fundamentally resolve the latency bottleneck. Flow Guided Policies (Jung et al., 2025) replaces the traditional iterative denoising process with a flow matching architecture. By modeling a continuous vector field, this method significantly reduces the required sampling steps and overcomes the limitations of open-loop execution commonly encountered in contact-rich tasks. In physical experiments, this vector-field architecture allows robots to achieve smoother, high-frequency action steering during contact-intensive manipulation, presenting clear utility for real-world physical deployments. Extending these real-time capabilities to highly dynamic platforms, DiffuseLoco (Huang et al., 2025) demonstrates that optimized diffusion models can meet the strict latency requirements of legged locomotion. By achieving 100 Hz inference for torque-level control, this approach successfully deploys multimodal, terrain-adaptive gaits across both quadrupedal and bipedal robots without compromising stability.

#### 3.4.3 Data efficiency

While improving inference speed remains essential, attaining operational viability in physical environments necessitates optimizing data-efficient training paradigms and reduced annotation dependence in training data.

MDT (Reuss and Lioutikov, 2023) integrates multimodal Transformer architectures with pre-trained foundational models to effectively learn long-horizon manipulation tasks from sparsely language-annotated game environments. This approach substantially reduces dependency on manual linguistic annotation for robotic manipulation tasks, thereby charting new pathways for developing multimodal policy frameworks in next-generation robotic learning systems.

Equivariant Diffusion Policy (Wang D. et al., 2024) introduces the concept of equivariance into diffusion policies, using domain symmetry to improve training sample efficiency and the generalization ability of diffusion models. This work theoretically proves that denoising diffusion functions are equivariant under certain conditions and demonstrates the application of SO(2)-equivariance in 6-Degree-of-Freedom (6-DoF) control for robotic manipulation. The authors then propose a new architecture that leverages equivariant networks to train the diffusion model and generates target action sequences through decoding with equivariant embeddings. Equivariant Diffusion Policy addresses sample intensity bottlenecks by enforcing geometric constraints, successfully allowing robots to handle cluttered real-world picking tasks after training on fewer than 50 demonstrations, a critical advancement for data-efficient physical manipulation.

#### 3.4.4 Hierarchical and structural optimization

To overcome the limitations of long-horizon tasks and complex action spaces, researchers have introduced hierarchical architectures and auxiliary supervision mechanisms. HDP (Ma et al., 2024) exemplifies this approach by combining a high-level next-best-pose agent with a low-level goal-conditioned diffusion policy. By utilizing differentiable dynamics to ensure kinematic compliance, HDP achieves an 80.18% success rate across 11 RLBench tasks, significantly outperforming traditional hierarchical baselines like PerAct + Planner.

For complex long-horizon tasks, hierarchical frameworks like DISCO (Hao et al., 2024) and Track2Act (Bharadhwaj et al., 2024) leverage keyframes or high-level plans derived from visual language models and internet videos to guide the diffusion process, facilitating general robot manipulation in complex scenes. Beyond hierarchy, structural optimization through self-supervised objectives, such as the state decoder in Crossway Diffusion (Li et al., 2024), enhances intermediate representations, providing a more robust mapping between high-dimensional observations and low-level control while preventing representation collapse.

Expanding beyond stationary platforms, traditional imitation learning has established a strong foundation for transferring agile behaviors in legged locomotion (Mirza and Singh, 2025); however, conventional methods often struggle with multimodal human demonstrations and highly unstable dynamics. Diffusion models address these limitations by effectively modeling complex action distributions while satisfying strict physical constraints. Building on these capabilities to address the unique challenges of mobile manipulation, recent works such as (He et al., 2024) introduce hierarchical diffusion frameworks for quadrupedal loco-manipulation. By employing a diffusion-based behavioral cloning planner at the high level, this architecture seamlessly coordinates end-effector manipulation tasks (e.g., door pushing) with underlying legged mobility.

#### 3.4.5 Spatiotemporal and 3D representation learning

A critical shift in imitation learning involves transitioning from 2D pixel-based inputs to high-dimensional 3D and spatiotemporal representations, explicitly encoding spatial affordances. DP3 (Ze et al., 2024) pioneeringly integrates 3D visual representations into diffusion policies, demonstrating that compact point cloud encodings provide superior environmental priors for complex, high-dimensional tasks with minimal demonstrations. The utility of 3D representations has been validated in physical experiments involving deformable object manipulation and cluttered bin picking. Building on this, 3D Diffuser Actor (Ke et al., 2024) and DNAct (Yan et al., 2024) utilize 3D scene representations and NeRF-based pretraining to distill common-sense semantic features into the policy manifold, enhancing zero-shot transfer capabilities for real-world physical tasks. While DP3 leverages raw point clouds for geometric and sample efficiency, 3D Diffuser Actor and DNAct represent a functional evolution by embedding rich semantic priors, illustrating a trade-off between computational lightweightness and semantic generalization.

Complementing these explicit 3D structures, R&D (Vosylius et al., 2024) unifies robotic motions and RGB observations directly within a shared image space, allowing policy actions to align strictly through the generative diffusion process. This rendering-based representation provides an elegant alternative that bypasses explicit 3D grid layouts while maintaining robust execution across RLBench benchmarks and physical platforms at the cost of increased dependency on uniform camera viewpoints. This highlights a fundamental architectural divergence in the field: explicitly constructing 3D/NeRF spaces offers robustness against viewpoint shifts, whereas implicit 2D rendering prioritizes computational efficiency under constrained camera setups.

Recent advancements have further extended spatial understanding into the temporal domain through four-dimensional spatiotemporal perception. ISS Policy (Xia et al., 2025) addresses the over-reliance on object appearance by aligning policy outputs with the geometric evolution of the scene, thereby improving generalization to unseen structures. Advancing this paradigm, DP4 (Liu et al., 2025) constructs a dynamic 4D Gaussian world model that explicitly encodes spatiotemporal changes into the diffusion process. This design allows robots to anticipate environmental dynamics, enabling precise control in interactive settings involving dynamic objects. The transition from static 3D (e.g., DP3) to dynamic 4D representations resolves a major limitation of earlier methods: the inability to natively model environmental reactivity over time.

Furthermore, to ensure operational robustness in these high-dimensional spaces, frameworks like DisDP (Vanjani et al., 2025) employ disentangled representations to maintain decision stability despite sensor noise or severe occlusions characteristic of complex simulated environments. DisDP’s approach underscores a critical deployment reality: as representation dimensionality scales from 2D to 4D, the policy’s susceptibility to sensor noise grows proportionally, making representation disentanglement a mandatory regularization step rather than an optional enhancement.

#### 3.4.6 Skill generalization and embodied foundation models

To achieve broad task generalization and human-aligned performance, the research community has moved toward skill-conditioned policies and large-scale foundation models, bridging the embodiment gap. XSkill (Xu M. et al., 2023) replaces rigid goal-conditioned targets with reusable skill prototypes learned from unlabeled human videos, facilitating cross-embodiment skill transfer for complex kitchen tasks. To align these generative outputs with dynamic human intentions, FDPP (Chen Y. et al., 2025) incorporates reinforcement learning with human feedback to continuously fine-tune pretrained diffusion models. This ensures that generated trajectories remain consistent with human intuition and safety requirements in simulated interactive environments. Additionally, specialized tasks such as navigation and collaborative control, addressed by NoMaD (Sridhar et al., 2024) and (Yoneda et al., 2023b), demonstrate the versatility of diffusion policies in switching between exploration and goal-oriented behaviors across diverse terrains.

The culmination of these efforts is the development of embodied foundation models that overcome hardware heterogeneity. RDT (Liu et al., 2024) introduces a diffusion foundation model for dual-arm manipulation, utilizing a Diffusion Transformer (DiT) backbone to unify multimodal inputs including vision, language, and proprioception. By projecting diverse robotic action spaces into a physically interpretable unified manifold and pretraining on over one million trajectories, RDT achieves unprecedented cross-task generalization. Specifically, RDT has been successfully deployed on physical dual-arm platforms in residential environments, performing high-precision chores with heterogeneous hardware. Complementing this, DiffusionVLA (Wen et al., 2025) integrates autoregressive reasoning with continuous action generation, providing explicit reasoning traces that enhance the interpretability and success rates of multi-stage operations, thereby bridging the gap between high-level semantic reasoning and autonomous physical execution while mitigating black-box unpredictability.

### 3.5 Task planning and reasoning

In addition to low-level control, diffusion models exhibit exceptional capacity for high-level task reasoning when leveraging representation scaling on rich datasets. These models function as hierarchical planners that decompose abstract instructions into structured subgoals or intermediate keyframes. Unlike symbolic planners that rely on predefined logic, diffusion-based methods treat task planning as a generative process, modeling the probabilistic relationships between environmental states and intended outcomes. This approach is particularly suitable for long-horizon tasks that require anticipating constraints and managing environmental uncertainty. By framing planning as a conditional denoising process, these models connect semantic understanding with physical execution, facilitating autonomous behavior in unstructured environments. A categorization of diffusion models in task planning and reasoning is provided in Table 4.

**TABLE 4:** A categorization of diffusion models in task planning and reasoning, highlighting technical innovations in geometric motion planning, hierarchical skill composition, and visual reasoning within the current robotic research landscape.

Selected works	Year	Target problem	Contribution	Simulation platform and dataset
(Carvalho et al., 2022)	2022	Motion Planning	Proposing conditioned score-based models for robotic motion planning tasks in complex environments	RECTANGLES AND CIRCLE
SE(3)-DiF (Urain et al., 2023)	2023	Motion Planning	Proposing a unified optimization framework for learning smooth cost functions to generate motion trajectories in SE(3) space	IsaacGym; Real-world
MPD (Carvalho et al., 2023)	2023	Motion Planning	Learning prior trajectory distributions using diffusion models	PointMass2D dense; PointMass3D maze boxes
DiMSam (Fang et al., 2024)	2024	Motion Planning	Generating constraint-satisfying action sequences and corresponding parameters for each action using diffusion model	IsaacGym; Real-world
(Power et al., 2023)	2023	Motion Planning	Initializing the constrained stein Variational trajectory optimization with trajectory distributions generated by diffusion models	12-DoF quadrotor task; 7-DoF robot manipulator
EDMP (Saha et al., 2024)	2024	Motion Planning	Learning latent potentials and composing them as a cost function enabling generalization to various motion constraints	Pybullet simulator; M π Nets
(Luo et al., 2024)	2024	Motion Planning	Learning latent potentials and composing them as a cost function enabling generalization to various motion constraints	Maze2D; KUKA; Dual KUKA; Real-world
StructDiffusion (Liu et al., 2023b)	2023	Task planning; Rearrangement	Generating plausible object arrangement scenes using diffusion models to guide rearrangement tasks	PyBullet simulator; Real-world
DALL-E-Bot (Kapelyukh et al., 2023)	2023	Task planning; Rearrangement	Applying web-scale network diffusion models to task planning for guiding robotic rearrangement tasks	Real-world
ReorientDiff (Mishra and Chen, 2024)	2024	Task planning; Rearrangement	Employing a diffusion model to sample intermediate poses from a joint representation, making rearrangement more precise	PyBullet simulator; OMPL
SetItUp (Xu et al., 2024b)	2024	Task planning; Rearrangement	Harnessing LLMs to generate abstract spatial relationships between objects as constraint conditions, handling ambiguous instructions	Rearrangement on study desks Dining tables, and coffee tables
RPDiff (Simeonov Et Al., 2023)	2023	Task planning; Rearrangement	Formulating rearrangement task as point cloud denoising process by learning geometric relationships between objects and scenes	PyBullet simulator; Real-world
Diffusion-CCSP (Yang et al., 2023c)	2023	Task planning	Representing constraints as factor graphs by training diffusion models for every constraint and compositing them	Four multi-constraint domains
GSC (Mishra et al., 2023)	2023	Task planning	Formulating robotic task as a skill chain, where diffusion models are trained for individual skills and assembled during inference	Toy domain; PyBullet simulator
Planning as In-painting (Yang et al., 2023a)	2023	Task planning	Formulating task planning as an image inpainting problem to jointly estimate target states and trajectories	CompILE; Kuka robot; ALFRED
Diffusion-EDFs (Ryu et al., 2024)	2024	Task planning	Developing a bi-equivariant diffusion model on the SE(3) manifold for task planning, which enhances sample efficiency	Sapien; Real-world
SuSIE (black et al., 2024b)	2024	Task planning; Text-image	Employing a text-image diffusion model to generate future frame from current frames and task descriptions for task guidance	Calvin; BridgeData V2
GENIMA (shridhar et al., 2024)	2024	Task planning; Text-image	Rendering joint target positions and rotation angles as colored spheres on the image to guide generation of joint position sequences	CoppeliaSim; RLBench; Real-world
CoTDiffusion (Ni et al., 2024)	2024	Task planning; Text-image; Hierarchical	Utilizing the diffusion model as a high-level visual planner to generate semantically aligned keyframes for visual guidance	VIMA-BENCH
UniPi (Du et al., 2023b)	2023	Task planning; Text-Video	Training a text-video diffusion model to generate highly realistic video plans to guide robotic action execution	PDSketch; Cliport; Real-world
AVDC (Ko et al., 2024)	2024	Task planning; Text-Video	Estimating optical flow between adjacent frames in synthetic videos utilizing optical flow and initial frame depth to estimate actions	Meta-World; iTHOR; Bridge; Real-world
HiP (Ajay et al., 2023b)	2023	Task planning; Text-Video	Using LLMs to decompose language instructions into subgoal sequences, guiding the generation of video diffusion models	Paint-Bl0ock; object-arrange; Kitchen-tasks
VLP (Du et al., 2023a)	2023	Task planning; Text-Video	Using VLMs to generate next-step action descriptions, combined with parallel hill climbing to enhance video reliability	Language table; Real-world
RoboDreamer (Zhou et al., 2024b)	2024	Task planning; Text-Video	Parsing raw instructions into low-level language units and generating video plans based on these units	RLBench; RT-1
This&That (Wang et al., 2024a)	2024	Task planning; Text-Video	Introducing additional gesture cues on initial frames to deliver targeted guidance for diffusion model	Bridge; IsaacGym
SkillDiffuser (Liang et al., 2024)	2024	Task planning; Text-Video	Encoding skills into finite skill sets via Vector quantization and utilizing skill abstractions as conditioning inputs for diffusion models	LOReL sawyer; Meta-world
(Botteghi et al., 2023)	2023	Motion planning; Safety	Training a DDPM with CBF constraints for trajectory safety classification and security evaluation	OpenAI gym
SafeDiffuser (Xiao et al., 2025)	2025	Motion planning; Safety	Integrating CBFs into conditional generation process of diffusion model to enforce generated trajectory safety	D4RL
(Lee et al., 2023)	2023	Motion planning; Safety	Proposing a new recovery gap metric to evaluate the feasibility of plans generated by diffusion models	Maze2D; Gym MuJoCo; Block stacking
LTLDoG (Feng et al., 2024)	2024	Motion planning; Safety	Incorporating LTLf into the diffusion model to ensure generated trajectories satisfy specified static and temporal constraints	Maze2D; Push-T; Real-world
CAMPD (Sandra et al., 2025)	2025	Motion planning; Acceleration	Leveraging context-conditioned diffusion models to accelerate multimodal trajectory generation	M π Net; Sphere-based
MoE DP (cheng et al., 2025)	2025	Task planning; Skill chaining	Integrating a mixture of experts architecture for dynamic skill decomposition and autonomous failure recovery	MimicGen; Real-world
DiffusionVLA (Wen et al., 2025)	2025	Task planning; Foundation	Unifying autoregressive reasoning with diffusion models to output explicit reasoning traces for multi-stage operations	OXE; Droid; Real-world
HARMON (Jiang et al., 2025)	2024	Semantic reasoning; Whole-body control	Iteratively evaluating and refining diffusion-generated humanoid motions using Vision-language models as commonsense reasoners	Fourier GR1; Real-world
(Taouil et al., 2025)	2025	Visual planning; Whole-body control	Synthesizing 2D interaction scenes via LDMs to extract explicit 3D keyframes, guiding whole-body trajectory optimization solvers	MuJoCo; Unitree G1

#### 3.5.1 Geometric and motion planning

(Carvalho et al., 2022) are the first to propose conditioned score-based models for robotic motion planning tasks in complex environments. This work utilizes signed distance functions to encode the geometric properties of environmental maps, demonstrating promising performance in 2D navigation tasks, and laying the foundation for applications in robots with higher degrees of freedom. SE(3)-DiF (Urain et al., 2023) learns a smooth cost function in SE(3) space and trains the diffusion model through joint gradient optimization, achieving outstanding performance in 6-DoF grasping tasks. MPD (Carvalho et al., 2023) also utilizes a diffusion model to fit trajectory distributions and demonstrates its effectiveness in 3D maze navigation and 7-DoF robotic manipulation tasks. DiMSam (Fang et al., 2024) employs diffusion models to generate action sequences that adhere to constraints along with parameter values for each action, partially addressing the challenge of multi-step robotic operations in partially observable environments.

(Power et al., 2023) utilize the trajectory distribution generated by diffusion models as an initialization for Constrained Stein Variational Trajectory Optimization, demonstrating generalization to unseen constraint combinations during training in a 12-DoF quadrotor task. This framework’s ability to navigate high-dimensional autonomous systems in complex environments confirms the potential of diffusion models for agile robotics. EDMP (Saha et al., 2024) also uses a diffusion model to learn motion trajectory patterns. Their method proposes an Ensemble-of-Costs Guidance technique. This guidance helps generate trajectories that meet specific needs by combining different cost functions. (Luo et al., 2024) propose Potential-Based diffusion motion planning, which learns and combines potential fields to achieve generalization across diverse motion constraints. The feasibility of this approach has been validated on physical KUKA manipulators, where it successfully guides robotic movements through dense obstacle clusters. To address the intrinsic sampling efficiency bottleneck, CAMPD (Sandra et al., 2025) leverages context-conditioned diffusion models to directly learn multimodal data distributions. By conditioning the generative process on sensor-agnostic contextual information, this approach significantly accelerates the generation of low-level trajectories that satisfy complex physical constraints. This acceleration provides highly efficient foundational support for high-level logical planning without requiring environmental retraining, albeit at the cost of requiring high-fidelity contextual priors.

#### 3.5.2 Semantic reasoning

In order to enable robots to autonomously execute complex high-level tasks, task planning is indispensable. In recent years, the application of diffusion models in task planning has also garnered significant attention.

The implementation of diffusion models for task planning first materialized in the domain of object reorientation, where StructDiffusion (Liu W. et al., 2023) employs robots to accomplish object rearrangement tasks. This approach inputs point clouds and language instructions into diffusion models, enabling the rearrangement of unseen objects into physically plausible structures, thereby guiding the motion planning. The real-world utility of StructDiffusion has been demonstrated in complex tabletop rearrangement tasks with novel items, bridging the gap between semantic instruction and physical orientation. DALL-E-Bot (Kapelyukh et al., 2023) employs a web-scale trained diffusion model for task planning, generating target images through natural language descriptions, which then guide robots to rearrange objects via grasping and placing operations. However, this work is only applied to 3-DoF rearrangement tasks. Subsequent research extends it to higher DoF object rearrangement tasks.

In order to enable robots to autonomously execute complex high-level tasks, task planning is indispensable. In recent years, the application of diffusion models in task planning has also garnered significant attention.

The implementation of diffusion models for task planning first materialized in the domain of object reorientation, where StructDiffusion (Liu W. et al., 2023) employs robots to accomplish object rearrangement tasks. This approach inputs point clouds and language instructions into diffusion models, enabling the rearrangement of unseen objects into physically plausible structures, thereby guiding the motion planning. The real-world utility of StructDiffusion has been demonstrated in complex tabletop rearrangement tasks with novel items, bridging the gap between semantic instruction and physical orientation. DALL-E-Bot (Kapelyukh et al., 2023) employs a web-scale trained diffusion model for task planning, generating target images through natural language descriptions, which then guide robots to rearrange objects via grasping and placing operations. However, this work is only applied to 3-DoF rearrangement tasks. Subsequent research extends it to higher DoF object rearrangement tasks.

ReorientDiff (Mishra and Chen, 2024) employs a diffusion model to sample intermediate poses from a joint representation, enabling robots to achieve more precise object placement in both position and orientation. These existing studies primarily focus on object reorientation under explicit instructions, which imposes high demands on command accuracy and specification details. SetItUp (Xu Y. et al., 2024) introduces a novel approach to interpret and execute ambiguous instructions. This framework utilizes LLMs to first generate abstract spatial relationships between objects as geometric constraints. Subsequently, it combines multiple diffusion models to generate object poses that satisfy these constraints. In addition, RPDiff (Simeonov et al., 2023) approaches rearrangement prediction as a point cloud pose denoising problem. This method learns geometric relationships between objects and scenes through point cloud inputs. It generates multiple potential rearrangement configurations. Physical experiments have shown that RPDiff enables robots to perform precise shelving and packing tasks in laboratory settings, highlighting the robustness of point cloud-based reasoning. However, this approach has limitations in requiring substantial training data. Obtaining 3D point clouds from real-world scenarios proves particularly challenging, which creates operational friction for deployment in highly dynamic real-world settings. Expanding semantic reasoning beyond static object rearrangement, HARMON (Jiang et al., 2025) introduces a framework for generating and refining whole-body humanoid motions from free-form language descriptions. By employing Vision-Language Models (VLMs) as commonsense reasoners, the system iteratively evaluates and edits the trajectories generated by a human-motion diffusion prior. This closed-loop semantic feedback allows the robot to accurately deduce fine-grained behaviors, such as specific finger gestures and head orientations, bridging the gap between high-level linguistic intent and complex kinematic execution.

#### 3.5.3 Constraint satisfaction and skill chaining

Executing sophisticated tasks requires robots to navigate a landscape of competing physical and environmental constraints, such as joint limits, singularities, and obstacle avoidance. The flexibility of diffusion models lies in their ability to treat these constraints as guidance signals or energy functions, enabling the composition of specialized skills into coherent, long-term plans through iterative refinement.

Diffusion-CCSP (Yang et al., 2023c) proposes a constraint satisfaction framework for robotic reasoning and planning. This method employs factor graphs to visually represent different constraints. Specifically, a diffusion model is trained for each constraint type and transformed into an energy function. The energy functions collectively form a global optimization objective. Through iterative energy minimization sampling, solutions satisfying all constraints can be systematically generated. GSC (Mishra et al., 2023) approaches robot task planning by constructing a skill chain. This method trains an unconditional diffusion model for each individual skill. During inference time, these models are sequentially combined to address unseen long-term goals. However, this approach has only been experimentally validated in fully observable environments, exhibiting performance degradation under stochastic states.

In contrast, Planning as In-Painting (Yang C.-F. et al., 2023) frames task planning as an image in-painting problem. This method simultaneously estimates target states and trajectories through joint optimization. The practical applicability of this work is evidenced by its successful deployment on physical KUKA manipulators for long-horizon assembly tasks, effectively addressing partial observation challenges. The proposed framework enhances algorithmic adaptability in complex environments by implementing dynamic plan updates during execution. Diffusion-EDFs (Ryu et al., 2024) proposes a bi-equivariant diffusion model based on the SE(3) manifold for task planning. This approach utilizes the invariance properties of translation and rotation. Such properties allow the model to be trained with limited data while maintaining generalization capabilities to unseen task configurations during testing. However, this study does not achieve control-level or trajectory-level reasoning. Equivariant Diffusion Policy later addresses this limitation.

While chaining individual skills provides a theoretical pathway to solve complex tasks, executing these sequences in dynamic environments requires robust adaptability to unexpected disruptions. To achieve this, MoE DP (Cheng et al., 2025) integrates a mixture of experts architecture into the diffusion policy framework. This approach enables end-to-end dynamic skill decomposition for long-horizon tasks. More importantly, it grants the robotic system the capacity for autonomous failure recovery. In real-world robotic manipulation experiments, the model demonstrated the ability to retry failed pick-and-place subtasks when encountering external disturbances, significantly enhancing the reliability of long-term constraint satisfaction for autonomous systems.

#### 3.5.4 Visual planning and video synthesis

While diffusion models have been widely applied in generating action sequences and parameterized motions, their primary strength still lies in image generation. Recent studies demonstrate that image diffusion models can effectively synthesize goal states for robotic tasks. More importantly, these models enable the decomposition of complex tasks into sequential modular operations. As shown in Figure 5, the emergence of diffusion models has made visual prompts a key tool in robotic task reasoning. Diverging from purely text-based prompts, closed-loop image prompts condition on camera-captured current frames to continuously generate subsequent frames upon completing the underlying control task. This closed-loop generative process enables dynamic task planning adjustments through iterative refinement cycles. Such a hierarchical approach has shown promising results in robotic manipulation scenarios, particularly in solving long-horizon tasks through stepwise visual planning.

**FIGURE 5:**
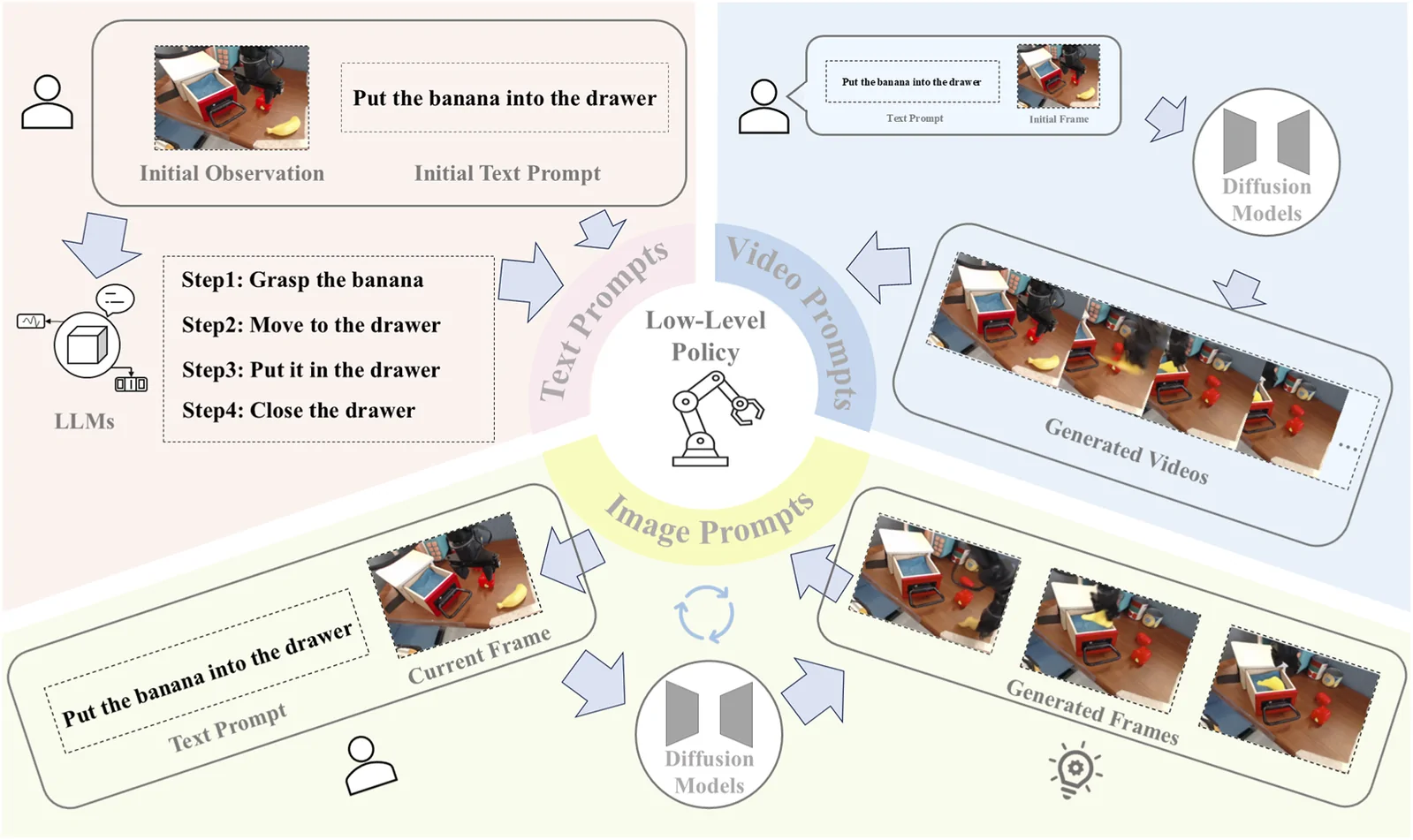
Comparative analysis of three task reasoning paradigms in robot learning. Large Language Models function as high-level planners by decomposing complex instructions into sequential text-based subgoals. In contrast, diffusion models synthesize high-fidelity video trajectories or iteratively generate intermediate keyframes from current observations and linguistic inputs. Unlike low-bandwidth text, these visual representations provide rich spatial-temporal context and superior representational capacity, significantly bolstering the agent’s ability to comprehend and execute multi-stage robotic tasks.

SuSIE (Black et al., 2024b) fine-tunes the InstructPix2Pix (Brooks et al., 2023) model to generate hypothetical future frames based on current visual frames and language descriptions. These frames serve as high-level guidance for task execution. SuSIE’s performance has been validated on the BridgeData V2 real-robot dataset, demonstrating its capability to guide physical robots through novel manipulation tasks with zero-shot generalization. A low-level controller then translates this guidance into concrete robot actions. By leveraging web-scale training data, the system demonstrates enhanced capabilities in recognizing and reasoning about novel objects and scenarios beyond those encountered in domain-specific datasets. The separation of high-level visual reasoning and low-level control enables effective generalization to unseen environments.

GENIMA (Shridhar et al., 2024) introduces additional information into generated images to enhance system performance. This study fine-tunes the Stable Diffusion model (Rombach et al., 2022) to predict future joint position targets based on input RGB images and linguistic objectives. The predicted joint positions are visualized as colored spheres superimposed on the images. These spheres contain horizontal stripes that explicitly indicate joint rotation angles through their spatial patterns. Experimental results demonstrate that this information fusion approach achieves superior persuasiveness and accuracy compared to single-modality guidance methods. CoTDiffusion (Ni et al., 2024) employs a hierarchical control approach for robotic manipulation, using diffusion models as high-level visual planners. This method features a semantic alignment module that iteratively generates sub-goal images for subsequent steps. These images are aligned with multimodal instructions to provide clear semantic guidance for the diffusion process. Through rigorous semantic alignment, CoTDiffusion produces coherent sub-goal image sequences that closely follow task requirements. Pushing visual planning into the realm of complex humanoid control, (Taouil et al., 2025) utilize Latent Diffusion Models (LDMs) to synthesize 2D RGB scenes depicting human-object interactions. By extracting precise 3D contact locations and kinematically feasible robot configurations directly from these generated images, the framework provides explicit visual keyframes for a whole-body Trajectory Optimization (TO) solver. This visually-guided planning approach successfully orchestrates long-horizon loco-manipulation tasks—such as pushing a trolley or retrieving elevated objects—without requiring heavily annotated 3D datasets.

UniPi (Du et al., 2023b) proposes a novel approach by transforming sequential decision-making problems into text-conditioned video generation tasks. This method employs pretrained language embeddings and leverages internet video datasets to synthesize highly realistic video plans. These generated video sequences serve as visual guidance for robot action execution. High-level planning based on videos provides more detailed guidance. The effectiveness of UniPi has been demonstrated in physical environments, enabling robots to perform diverse table-top manipulation tasks with high spatial consistency. However, it also has drawbacks, such as slow inference speed and the potential for generating unrealistic objects or motion hallucinations in partially observed environments.

To mitigate the gap between hallucinated video plans and actionable kinematics, AVDC (Ko et al., 2024) employs a video generation approach to guide task execution. This method specifically calculates optical flow between consecutive frames in synthesized videos, establishing pixel-level dense correspondences. By testing on the Bridge dataset, AVDC has proven that video-to-action transfer remains robust across diverse table-top setups. The system then combines this optical flow data with depth information from the initial frame. Through this integration, it determines the required robotic actions. By explicitly grounding generative videos in optical flow, AVDC demonstrates how spatial priors can prevent the kinematic failures often seen in pure image-to-action models.

Beyond low-level action extraction, subsequent research focuses on improving conditional inputs to diffusion models or incorporating additional information to enhance generation quality. For instance, HiP (Ajay et al., 2023b) employs two key components in robotic task planning. First, a video diffusion model performs visual planning of action sequences. Second, LLMs decompose language instructions into structured subgoal sequences that guide the video generation process. However, the subgoal sequences generated by LLMs remain static and fail to account for real-time robot states during task execution. In contrast, VLMs demonstrate dynamic reasoning capabilities by processing current state images as input. This enables continuous adaptation to environmental changes during operation.

Therefore, VLP (Du et al., 2023a) employs VLMs to generate subsequent text-based actions. VLP has been successfully deployed in the real-world Language Table environment, demonstrating that closed-loop video planning can significantly enhance robotic task success rates. Using VLMs as heuristic functions, the parallel hill climbing algorithm conducts a forward search in potential video sequence spaces, enhancing video generation reliability. Unlike text instruction generation methods, RoboDreamer (Zhou S. et al., 2024) parses raw commands into low-level language units and generates video plans based on these units. This approach also accommodates multimodal inputs (e.g., target images and sketches), providing richer information for objective generation. This&That (Wang B. et al., 2024) improves video generation specificity by introducing additional gesture information on initial frames. Validated on the Bridge robotic platform, this gesture-guided framework facilitates more intuitive and reliable video plan generation for human-robot interaction. Distinct from all aforementioned methods, SkillDiffuser (Liang et al., 2024) learns discrete, human-interpretable skill representations from visual observations and language instructions. It quantizes skills into a finite set through Vector Quantization, using skill abstraction as guidance for video diffusion models. Since the model does not directly utilize visual information and instructional data as guidance, it demonstrates enhanced generalization capability when handling diverse robot morphologies and ambiguous instructions. This evolution highlights a crucial paradigm shift: treating visual and video diffusion not merely as open-loop trajectory generators, but as dynamic, closed-loop reasoning engines tightly coupled with state feedback.

### 3.6 Other applications

In addition to the aforementioned applications, several studies have explored alternative pathways by applying diffusion models to novel research directions in robotics. The widespread adoption of diffusion models has significantly promoted technological advancements across multiple dimensions.

The placement and stacking of objects involve many interactions. However, robotic systems typically lack prior knowledge about geometric properties of objects. To address this challenge, recent studies have employed diffusion models to infer reasonable object poses for robotic manipulation tasks. These models provide precise guidance for object placement operations. For instance, the 6-DoFusion method (Yoneda et al., 2023a) utilizes diffusion models to predict interactive object poses. This approach specifically models object interactions by incorporating Signed Distance Field (SDF) values as supplementary inputs during training. The diffusion model processes both point cloud data and corresponding SDF values simultaneously. This dual-input strategy enables generation of stable 6-DoF poses that account for physical interaction constraints.

Many robotic manipulation tasks involve handling complex contact dynamics and geometric relationships. However, practical acquisition of tactile images under diverse object poses, positions, and contact conditions requires extensive experiment with precise control of environment. To address this challenge, (Higuera et al., 2023) developed a simulation-to-reality framework using diffusion models. Their approach generates realistic tactile images from simulated contact depth maps through an iterative denoising process. Experimental validation on braille reading tasks demonstrates significant advantages of the diffusion-based method. Compared with conventional techniques, the generated tactile images exhibit enhanced structural fidelity and finer textural details.

In addition to improving the ability to infer actions and trajectories, another effective approach to enhance task success rates involves addressing challenges through mechanical design. For specific tasks, customized grippers can be designed to reduce precision requirements in perception and control systems. The DGDM method (Xu X. et al., 2024) proposes a framework that decomposes manipulation tasks into sequential motion objectives called target interaction profiles. By comparing current and target interaction profiles, design objectives can be established to guide the geometric refinement of robotic fingers. However, this method employs oversimplified parameterization of actuators and stiffness properties, creating discrepancies between simulation results and real-world robotic performance. Future research could investigate more flexible parameterization approaches to bridge this implementation gap. DiffuseBot (Wang T.-H. J. et al., 2023) focuses on soft robot design challenges. This method first employs diffusion models to generate diverse 3D shapes as foundational robot geometries. The generated surface point clouds are then converted into robot-compatible representations incorporating material stiffness and actuator placement parameters. Finally, physical simulations are conducted to evaluate robot prototypes, enabling optimization of the diffusion model’s input embeddings. Through this co-optimization process, the method demonstrates enhanced robotic performance and successfully generates high-efficiency soft robot designs.

While video synthesis and visual planning provide intuitive guidance for robotic execution, these generative approaches primarily operate within the visual domain and often lack explicit logical deduction. For complex long horizon tasks, robots require not only visual foresight but also high level semantic understanding to make sequential decisions. Advancing beyond purely visual reasoning, the integration of diffusion models with large vision language models represents a critical frontier for developing embodied foundation models. This paradigm shift transitions the focus from generating pixel level plans to constructing unified cognitive architectures.

## 4 Discussion

After reviewing the relevant literature mentioned above, this section will focus on discussing the limitations and challenges. Some of these challenges are inherent to the field of robotics, and diffusion models show potential in addressing these issues. Based on these challenges and limitations, we provide prospects for future development in this field.

### 4.1 Safety issues

For robots to achieve autonomy, they must first overcome various safety challenges. However, due to imperfect dynamic models, inadequate handling of sensor measurement noise, and insufficient characterization of operating environments, robotic systems often exhibit uncertain dynamic behaviors. Although robots possess limited knowledge of the real world, they must still make reasonable decisions while ensuring behavioral safety, particularly in human-robot interaction tasks. Consequently, safety remains an unavoidable challenge in robotics.

Traditional approaches to addressing safety and adaptability challenges have evolved through two primary methodologies. Control theory-based methods employ predefined dynamical models to ensure system safety, yet their reliance on accurate physical parameters restricts environmental adaptability. Conversely, RL techniques adopt data-driven exploration to achieve environmental flexibility, but inherently compromise safety guarantees during the learning phase.

Recent research efforts have developed safety-focused solutions using robust model predictive control. These approaches explicitly model uncertain system dynamics while maintaining state constraints. In parallel, RL methods now incorporate exploration boundaries or safety verification layers to generate more conservative policy updates. The pioneering work CPO (Achiam et al., 2017) established constrained policy optimization in the Constrained Markov Decision Process framework, ensuring policy improvements while satisfying safety limitations. Recent research has integrated constraint formulations with diffusion models to improve safety in robotic control tasks. Botteghi et al. (Botteghi et al., 2023) developed a framework combining Control Barrier Functions (CBFs) and DDPMs. The system employs three separate DDPM modules: trajectory generation aligned with system dynamics, expected return estimation, and safety classification using CBF constraints. A novel sampling strategy incorporates both value functions and safety classifiers as guidance signals, enabling simultaneous optimization of task performance and constraint satisfaction. This architecture modularizes trajectory planning, value prediction, and safety verification components typically coupled in RL, significantly enhancing operational flexibility while maintaining safety guarantees. SafeDiffuser (Xiao et al., 2025) targets safety-critical trajectory planning in robotic navigation and manipulation tasks. This framework implements CBFs during the diffusion timesteps, embedding finite-time invariance properties into the denoising process to ensure probabilistic safety guarantees. Three specialized variants were developed to address different requirements: a constrained sampling module prevents local optima entrapment, an adaptive constraint relaxation mechanism balances safety-energy tradeoffs, and a hybrid optimization architecture preserves the diffusion model’s generative performance. These innovations enable simultaneous safety enforcement and trajectory quality maintenance through time-dependent barrier function integration.

(Lee et al., 2023) proposes a novel metric called recovery margin to quantify the feasibility of trajectories generated by diffusion models. This metric evaluates trajectory quality by measuring the degree to which noise-corrupted plans can be restored to their original states through denoising operations. The approach effectively filters out physically unrealizable trajectories, thereby enhancing operational safety in robotic systems. Concurrently, LTLDoG (Feng et al., 2024) embeds finite linear temporal logic (LTLf) formulas into the diffusion process to ensure trajectory compliance with spatiotemporal constraints. The framework implements two strategies: using LTLf formulas as conditional guidance during diffusion sampling, and training a verification network to predict constraint satisfaction probabilities for guiding denoising trajectory generation. Experiments demonstrate that LTLDoG successfully generates feasible trajectories meeting temporal logic requirements in both simulation environments and quadruped robot navigation tasks.

Looking forward, future research in diffusion based robotic safety must transcend *post hoc* filtering and move toward “Safety by Construction” within the generative manifold. A primary technical challenge lies in the integration of differentiable safety layers directly into the denoising steps. Instead of relying on external classifiers, future frameworks should investigate projected diffusion sampling, where each denoising iteration is mathematically projected onto a safe set defined by learned Lyapunov functions or analytical kinematic constraints. This would ensure that the generated trajectories are intrinsically safe throughout the entire reverse process rather than being corrected only at the final output.

Furthermore, the research community should prioritize uncertainty-aware safety mechanisms that leverage the probabilistic nature of diffusion models. Specifically, the variance across multiple denoising samples can serve as a high-fidelity proxy for epistemic uncertainty; in scenarios where the diffusion model exhibits high variance, the system should autonomously trigger a transition to a conservative, low-gain control mode to prevent physically dangerous actions in novel environments. Finally, resolving the latency-safety trade-off requires the development of safety-preserving distillation techniques. Future work should focus on consistency models that distill not only the expert behavior but also the underlying safety boundaries into a single-step policy. This ensures that even under the millisecond-level feedback requirements of dynamic hardware, the robot maintains a “safe-by-default” execution layer that remains robust to sensor occlusions and unpredictable actuator backlash.

### 4.2 Real-time inference and model size

The practical implementation of diffusion models in robotic systems is fundamentally constrained by the technical conflict between the high computational intensity of iterative denoising and the high-frequency requirements of real-time control. Unlike traditional discriminative policies that compute actions in a single forward pass, diffusion based methods require multiple sequential inferences to produce a single control signal. For large scale Vision Language Action (VLA) models such as RT-2 (Zitkovich et al., 2023) or RDT (Liu et al., 2024), the computational burden becomes prohibitive when deployed on edge hardware. For instance, a typical foundation model with billions of parameters may require several gigabytes of video random access memory, which often exceeds the capacity of embedded systems like the NVIDIA Jetson Orin or Xavier modules. In practical deployments, such as a mobile manipulator performing dynamic grasping, these large models often struggle to maintain an inference rate above one to 5 Hz. However, maintaining stable contact and reacting to environmental perturbations typically requires a feedback loop of at least 50 Hz. This mismatch leads to jerky movements and failure in tasks requiring high reactivity, illustrating that large scale models currently face a significant “execution gap” on physical platforms.

Beyond pure latency, energy consumption poses a severe limitation for battery-powered autonomous systems. The continuous execution of foundation scale diffusion models can drive power consumption to the upper limits of a mobile robot’s Thermal Design Power, leading to rapid battery depletion and thermal throttling. When a processor reaches its thermal threshold, it automatically reduces clock speeds to prevent hardware damage, resulting in an unpredictable drop in control frequency that can be catastrophic for robots in safety critical environments. To resolve these specific bottlenecks for foundation models, the research community is moving toward advanced distillation and quantization techniques. Specifically, Consistency Distillation allows for the compression of a heavy teacher model into a student policy capable of producing high quality actions in as few as one or two steps. Furthermore, adopting low precision numerical formats such as FP8 or INT8 can significantly reduce memory bandwidth pressure and energy cost per inference, making the deployment of large scale generative intelligence more sustainable.

Looking forward, achieving reliable and energy efficient deployment requires a transition toward more dynamic and integrated architectures. A critical frontier involves the implementation of adaptive step diffusion mechanisms, where the model autonomously modulates the denoising budget based on the instantaneous complexity of the task. In routine scenarios with low uncertainty, the system can terminate sampling early to conserve power, whereas in highly unstructured or contact rich environments, it can allocate more computational steps to ensure operational safety. Furthermore, the development of asynchronous multi scale hierarchies provides a viable pathway to accommodate large scale models by decoupling high level semantic reasoning from low level motor execution. In this paradigm, a large VLA model operates in a slow reasoning loop to handle task planning, while a lightweight, distilled student model resides in a high speed execution loop for precise control. This “slow-fast” architecture ensures that heavy generative reasoning does not compromise the reactivity of the low level controller.

Finally, the long term sustainability of generative intelligence in robotics depends on hardware algorithm co-design through hardware aware neural architecture search. Rather than porting generic models to robotic hardware, future research should focus on developing “robot native” backbones optimized for specific edge AI constraints such as memory bandwidth and specialized tensor cores. This approach includes the optimization of Sparse Attention mechanisms and Key-Value Cache management specifically designed for the temporal dependencies of robotic trajectory data. By aligning generative algorithms with the strict energy and latency budgets of physical hardware, the field can ensure that large scale foundation models remain computationally viable for long horizon autonomous missions in the real world.

### 4.3 Simulation to the real world gap

Acquiring real-world robotic datasets remains challenging due to high costs and technical constraints. Most current studies conduct experiments in simulated environments. This reliance creates the Sim2Real gap that significantly hinders embodied AI development.

The primary contributor to Sim2Real gap stems from disparities in physical modeling and environmental complexity. Current simulation platforms implement simplifications in dynamic properties, material characteristics, and scene configurations. These approximations render simulation-optimized approaches ineffective in physical deployments. Furthermore, real-world sensors exhibit distinct noise profiles that disrupt robotic perception systems. Additionally, computational latency in physical systems significantly exceeds simulation predictions, demanding more robust control algorithms to maintain operational stability.

To systematically address these challenges, recent research utilizes diffusion models across three primary dimensions: generative domain adaptation, structured knowledge transfer, and endogenous dynamics modeling.

Traditional domain randomization often fails to capture the high-dimensional visual complexity and sensor noise present in real-world environments. Generative domain adaptation addresses this limitation by using diffusion models as neural renderers. Instead of manually tuning simulation parameters, this approach reformulates Sim2Real as a conditional image-to-image translation task. Diffusion models leverage large-scale data priors to synthesize realistic textures, lighting, and sensor artifacts onto simplified simulation outputs. By training on these enhanced observations, robotic perception systems develop stronger resilience to the visual discrepancies encountered during physical deployment.

A significant development in this area is the transition from exact digital twins to the concept of digital cousins. The ACDC framework (Dai et al., 2024) demonstrates that maintaining semantic and geometric similarity is more critical for policy robustness than pursuing 1:1 physical replication. By generating diverse cousin environments from a single real-world image, diffusion models provide the distributional density required for effective training. This method allows agents to achieve high success rates in zero-shot transfer because the generative process aligns the training distribution with real-world complexity more effectively than traditional simulation paradigms.

Future research in generative domain adaptation is expected to prioritize two key frontiers: computational efficiency and temporal consistency. Current diffusion-based rendering suffers from high inference latency, necessitating the development of accelerated sampling techniques or consistency-distilled models for real-time, closed-loop applications. Furthermore, transitioning from static scene synthesis to dynamic, interactive generative world models remains a critical challenge. Future frameworks must ensure that synthesized environments respond to robotic actions with physical accuracy, moving beyond visual realism to provide a unified foundation for both perceptual and dynamical Sim2Real alignment.

Beyond visual synthesis, diffusion models serve as foundational priors that facilitate the transfer of structured knowledge across different domains. In this paradigm, pre-trained generative models act as a repository of spatial intelligence and intuitive physics derived from massive, unannotated video corpora. Frameworks such as Track2Act (Bharadhwaj et al., 2024) and ViLLA-X (Chen X. et al., 2025) demonstrate that these models can internalize the fundamental semantics of human-object interactions. By leveraging these generative priors, robotic agents can inherit a broad distribution of feasible trajectories, significantly reducing the sample complexity required for real-world adaptation. This shift allows the learning process to transition from a tabula rasa approach to one anchored by high-level behavioral common sense.

A critical technical component of this transfer involves latent space alignment to bridge the gap between simulation-trained policies and real-world dynamics. During deployment, the reverse process of the diffusion model acts as a structural regularizer for the agent’s actions. Techniques such as guided sampling allow researchers to steer simulated strategies toward high-density regions of the pre-trained real-world distribution. This synergy ensures that the robot’s output remains within physically plausible manifolds, even when the initial training occurred in a simplified or low-fidelity simulator. Consequently, these foundational priors effectively mitigate the distribution shift caused by disparate physical modeling and sensor noise.

Future research should prioritize cross-embodiment scaling to address hardware heterogeneity and explore hybrid architectures that combine symbolic reasoning with generative trajectories. Such integration is essential to improve the explainability and safety of transferred policies in complex, constrained environments.

Finally, generative world models offer an alternative to traditional physics engines by modeling robotic dynamics as learned distributions. Frameworks such as UniPi and PolyGRAD utilize diffusion processes to predict future states directly within the visual or latent manifold. By internalizing non-linear physical interactions through large-scale generative modeling, these systems circumvent the approximation errors inherent in simplified mathematical formulations, particularly for complex materials like fluids or textiles. This approach grounds robotic policies in high-fidelity dynamical representations rather than discretized simulation approximations.

A key priority for future work is achieving zero-shot generalization across environments with novel physical properties, such as varied friction or gravity. However, recursive generation often introduces compounding errors and physical drift, making long-horizon temporal stability a persistent challenge. To ensure the reliability of generative simulators in unstructured deployments, it will be essential to anchor these learned dynamics using sparse real-world feedback, thereby mitigating the divergence between synthesized predictions and physical reality.

### 4.4 Datasets and unified benchmarks

The scarcity of datasets remains a significant barrier in robot learning research. Although diffusion models can scale up robot manipulation datasets to some extent, they cannot generate realistic robot datasets with arbitrary scenes, objects, and diverse manipulation strategies. Researchers have begun developing large-scale datasets for robotics, such as Open X-embodiment (O’Neill et al., 2024), Robomind (Wu et al., 2024), and AgiBot World (AgiBot-World-Contributors et al., 2025). These datasets cover multiple robot platforms, tasks, and environments. However, several challenges persist. First, data structures lack standardization, and input modalities only partially include image, 3D vision, text, or tactile signals. In addition, incompatible formats between multi-robot datasets complicate data processing and loading. As computing power continues to grow and data accumulates, creating general-purpose pretraining models for robots has become an important research direction. This trend highlights the need for unified large-scale multi-robot datasets with standardized structures and formats, which remains a critical development goal in robotics.

Moreover, the limited availability of a consensus-based public benchmark in robotics significantly hampers fair and consistent performance evaluations. Although a variety of benchmarks and simulators have been developed to support advanced embodiments which include bimanual manipulators and quadrupeds, these platforms remain highly fragmented and lack a unified verification framework to consistently validate algorithmic efficacy. Consequently, standardized assessment metrics remain underdeveloped in this field. Although task success rates serve as a common evaluation criterion, they fail to quantify crucial capabilities including cross-environment adaptability, viewpoint generalization, task transferability, and safety. To address these gaps, establishing unified evaluation protocols across diverse robotic platforms and tasks represents an essential direction for robotic research, which will better support technological advancements in this domain.

### 4.5 Embodied foundation models

Foundation models trained on massive and diverse datasets demonstrate strong generalization capabilities and cross-task adaptability. While foundation models have achieved relative maturity in NLP and CV domains, significant developmental potential remains in robotics applications, where the “scaling law” is hindered by the lack of high-quality, physically-grounded data.

Recent research efforts have emerged to develop cross-environment, cross-task, and cross-embodiment foundation models. The primary challenge arises from robotic data heterogeneity, where divergent platform specifications, varying degrees of freedom (DoF), and inconsistent control frequencies result in significant action label discrepancies. To address this, RT-X (O’Neill et al., 2024), Octo (Mees et al., 2024), and Open-VLA (Kim et al., 2024) have adopted discretization and normalization techniques to map heterogeneous actions into a unified token space. However, this discrete tokenization often sacrifices the high-precision requirements of dynamic control and fails to ensure action-space interpretability, leading to potential maneuver conflicts. More recently, RDT (Liu et al., 2024) and π 0 (Black et al., 2024a) have shifted toward continuous diffusion-based backbones (e.g., Diffusion Transformers), which aggregate heterogeneous action spaces while preserving the multi-modal nature of robotic control. While these models improve generalization, they still rely on manually constructed action mappings which are difficult to scale across radically different embodiments, such as quadrupedal versus bipedal systems.

To bridge the gap between abstract reasoning and physical execution, future research must move beyond manual action space alignment and prioritize the following technically grounded directions.

First, the development of Physics-Informed Action Manifolds is essential. Instead of mapping actions to arbitrary tokens, future foundation models should learn to project heterogeneous control signals into a unified latent space anchored by physical units (e.g., torque, force, and velocity). This would allow the model to understand the dynamic implications of an action across different embodiments, ensuring that a “grasp” command translates correctly regardless of the specific actuator’s hardware constraints. Second, research should investigate Autonomous Semantic Action Discovery, utilizing LLMs to automatically categorize and align action primitives from unlabeled multi-robot datasets. This would resolve the interpretability crisis by providing semantic “traces” for each generative step, allowing for explicit reasoning over action conflicts in complex multi-robot collaborative environments.

Finally, a critical frontier lies in Cross-Modal Tactile-to-Action Grounding. Current foundation models are predominantly vision-centric; however, true cross-embodiment generalization requires integrating high-frequency tactile and force feedback. Future architectures should leverage multi-modal diffusion transformers to align visual objectives with haptic realities, enabling foundation models to adapt their control policies in real-time to the unpredictable friction and contact dynamics of the physical world.

Overall, this paper systematically reviews the application of diffusion models in robotic systems and comprehensively analyzes current research advancements. We first outlines the fundamental principles of diffusion models and their recent technological progress. Subsequently, the paper categorizes the functional roles of diffusion models across five primary domains: scaling up robotics data, RL, IL, task planning and reasoning and other applications. These applications effectively address persistent challenges in robotics such as insufficient training data, limited action modalities, and weak generalization capabilities. The concluding section identifies both existing challenges in robotics and limitations in applying diffusion models, particularly regarding safety concerns, real-time processing requirements, simulation-to-reality gaps, embodied foundation models, and the lack of standardized benchmarks. These unresolved issues present crucial research opportunities for future investigations. The study emphasizes that advanced generative models like diffusion architectures possess significant potential to accelerate robotic innovation.
